# Backcrossing Modulates the Metabolic Profiles of Anthocyanin-Pigmented ‘Vitamaize’ Lines Derived from Elite Maize Lines

**DOI:** 10.1007/s11130-024-01155-0

**Published:** 2024-02-09

**Authors:** Héctor Arturo Peniche-Pavía, Tzitziki González-Rodríguez, Axel Tiessen, Silvero García-Lara, Robert Winkler

**Affiliations:** 1 Cinvestav Unidad Irapuato and UGA-Langebio Irapuato, Km. 9.6 Libramiento Norte Carr. Irapuato-León, 36824 Irapuato, Gto. Mexico; 2 Present Address: Cinvestav Unidad Mérida, Department of Marine Resources, 97310 Mérida, Yuc. Mexico; 3 Tecnológico de Monterrey, School of Engineering and Sciences, EIC, Ave. Eugenio Garza Sada 2501, 64849 Monterrey, NL Mexico

**Keywords:** Direct mass spectrometry, Anthocyanins, Breeding, Carotenoids, High-quality maize

## Abstract

Vitamaize lines (VMLs) were created by backcrossing the pigmented aleurone trait into Centro Internacional de Mejoramiento de Maíz y Trigo (CIMMYT) maize lines (CMLs). This study evaluates metabolic differences between the VMLs and their original CMLs. Direct infusion mass spectrometry (DIMS) analyses, carotenoid profiling, total anthocyanins content (TAC) determination, and biochemical evaluation of the quality protein maize (QPM) endosperm trait allowed a comprehensive chemical characterization of the maize lines. DIMS data indicate higher hexoses and trigonelline content for most VMLs; the carotenoid profile revealed a decrease in β-cryptoxanthin to less than half of the original parent content for two VMLs but an augmentation for one VML. The pigmented aleurone VMLs did not inherit the complex QPM endosperm trait of the QPM CMLs. Except for anthocyanin accumulation, no other metabolites were consistently modified across all the backcross-generated maize lines with a pigmented aleurone trait. These findings suggest using genetic or metabolic markers rather than morphological or visual traits for future breeding programs.

**Supplementary Information:**

The online version contains supplementary material available at 10.1007/s11130-024-01155-0.

## Introduction

The consumption of blue corn and other foods rich in anthocyanins is booming for their positive effects on the intestinal microbiome [1], dyslipidemia [2], and cardiovascular system [3]. In recent years, plant breeders have generated elite lines of blue maize due to increased public interest in its sweeter taste, softer texture, and high concentration of antioxidants, specifically anthocyanins [4, 5]. Breeding anthocyanin-rich maize was mainly focused on yield, omitting molecular markers for the nutraceutical content [6]. Blue maize owes its color to the anthocyanin accumulation in the outer layers of the kernel, which are the pericarp and the aleurone, the outermost endosperm layer [7]. Anthocyanins are glucosides of anthocyanidins (flavylium-base flavonoids) that in maize can be mono or di-acylated in the hexose residue [8, 9].

Conventional breeding procedures like backcrossing are still essential, especially in countries like Mexico, which has a long history of bans on genetically modified (GM) crops, especially maize [10]. This study evaluated new inbred lines of pigmented maize created by backcrossing. The Centro Internacional de Mejoramiento de Maíz y Trigo (CIMMYT) maize lines (CMLs) were the recurrent parent, and a pigmented native maize ‘Xoxocotla’ (named after the harvest site) was the donor of the blue corn phenotype [11]. The plant improvement project objective was to introduce the pigmented-aleurone trait into the genetic background with better agronomical and nutritional characteristics, such as the quality protein endosperm (QPM) [12], phenolic antioxidants, and provitamin A [13]. Provitamin A refers to carotenoids, which are converted in the body to vitamin A, mainly β-carotene and β-cryptoxanthin. Also, non-provitamin A carotenoids in maize, such as lutein and zeaxanthin, contribute to human health by acting as antioxidants and immune system regulators [14, 15]. The new pigmented lines were named 'Vitamaize lines' (VMLs). After nixtamalization, the aleurone pigmentation produces anthocyanin-colored dough (masa) to elaborate colorful maize-based food (tortillas and tamales, among others) [16]. It is hypothesized that the backcrossing would produce not only anthocyanin accumulation but, in addition, metabolic modifications shared across all the developed pigmented maize lines.

A direct-liquid-infusion mass spectrometry (DIMS) metabolomic approach was implemented to explore the chemical differences that produced the backcrossing at the grain level [17]. This method skips a prior chromatographic separation; thus, a sample extract is infused directly into a mass spectrometer. The concept is named by multiple acronyms such as the International Union of Pure and Applied Chemistry (IUPAC)-recommended ‘DIMS’ [18], flow-injection electrospray (FIE)-MS [17], direct-injection electrospray ionization (DIESI)-MS [7], and direct liquid-injection electrospray (DLI-ESI)-MS [19]. The data quality of this approach allowed classifying maize samples between pigmented and non-pigmented grains [7], to characterize the acylation loss in the anthocyanin profile after lime processing on pigmented maize kernels [16], and to identify maize quantitative trait loci (QTLs) for maize and seed metabolites in the intermated B73 × Mo17 (IBM) population [20].

Pigmentation at the aleurone tissue happens due to the conjunction with three transcription factors that form the MYB-bHLH-WD40 repeat (MBW) transcriptional complex up-regulating the expression of the anthocyanin enzymatic machinery. The genes that are specifically involved in the accumulation of anthocyanins in maize aleurone are *c1* (*ZmMYB1*), *r1* (*ZmbHLH1*), and *pac1* (*ZmWD40*) [21]. In maize genotypes that present their colorless aleurone, it is mainly due to recessive alleles or mutations of the *c1* or the *r1* genes [22]. Therefore, this study includes an analysis of the origin of the *c1* and *r1* alleles in the VMLs.

## Materials and Methods

The material and methods section is included as Supplementary Material 1.

## Results and Discussion

### Pigmentation Patterns of CMLs and VMLs

**Fig. 1:**
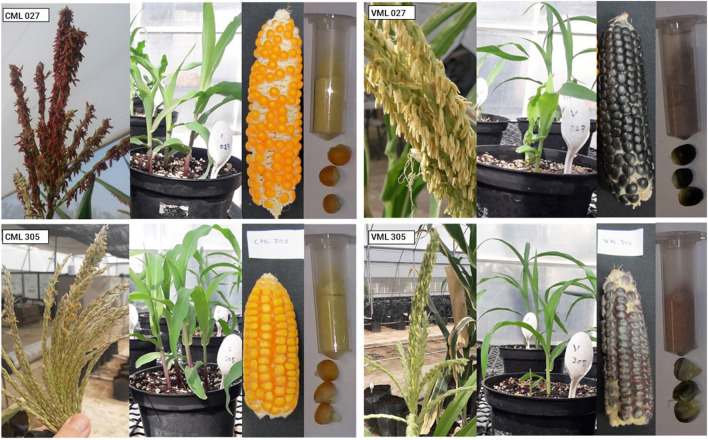
Pigmentation differences among the CMLs and VMLs. Contrary to CMLs, VMLs do not accumulate anthocyanins at the aleurone level. However, they have pigmentation at the anthers, bracts, and seedling sheaths, which could be related to the introgression of allele R-g in the VMLs after backcrossing. The VML 305 showed a red powder instead of a purple-colored powder like the rest of the VMLs

The group of CMLs comprises five genotypes with yellow endosperm (CML 027, CML 305, CML 327, CML 451, and CML 496) and five with white endosperm (CML 321 and CML 494, also having three QPM-type genotypes: CML 490, CML 491 and CML 492). The VMLs group presents the ten descendants obtained from the backcrossing process with a pigmented tester. The selected CMLs share pigmentation in the plant vegetative tissue. In particular, the bracts, anthers, and leaf sheath of seedlings (Fig. 1).

However, there is no pigmentation in the CML aleurones. The VMLs displayed a reverse scheme of pigmentation (see Fig. 1 and Figures S1-S11). The pigmentation patterns suggest that the pigmented donor Xoxocotla's complex MBW alleles were introduced into the CML genome during backcrossing [22]. Figure 1 shows also the final color of the ground sample.

### Concentration of Carotenoids and Anthocyanins in CMLs and VMLs

**Fig. 2:**
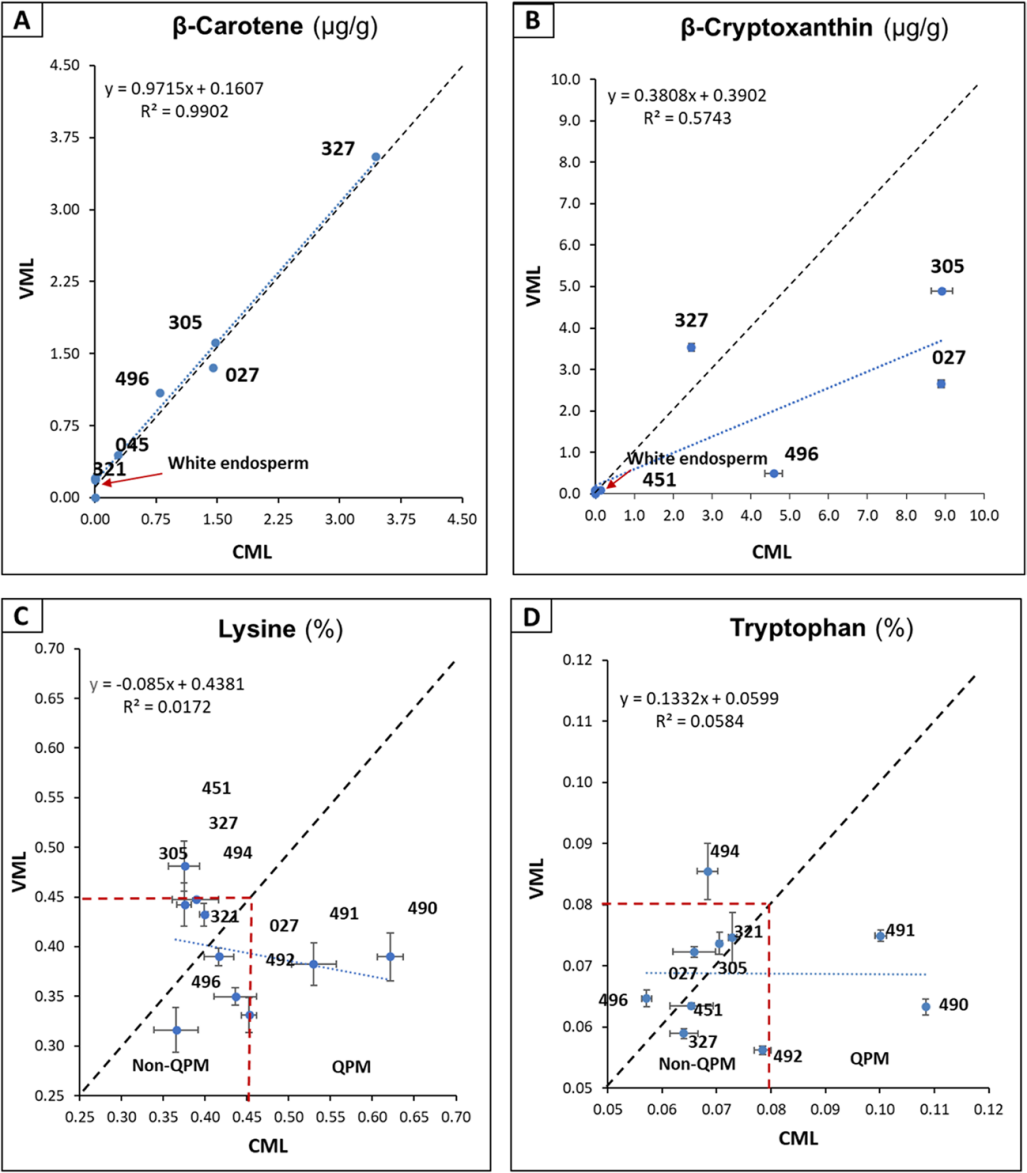
Comparison of β-carotene, β-cryptoxanthin, tryptophan, and lysine content between VMLs and CMLs. The black dashed line corresponds to a hypothetical correlation of one, i.e., no changes between VMLs and CMLs. The blue dashed line corresponds to the experimental trend line. The β-carotene content was inherited in the VMLs, while the carotenoid β-cryptoxanthin decreased significantly in the VMLs. The red dashed line corresponds to the QPM levels. The CMLs 490–492 are QPMs, but their descendants are not

The carotenoid concentrations of VMLs 027 and 496 decreased by up to 60% (See Table 1). In contrast, VML 327 and VML 451 increased their concentration of carotenoids. VML 327 increased its carotenoid concentration by 9% and VML 451 by 50%. Most of the VMLs from a white CML increased their concentration of carotenoids. However, their concentrations were less than one µg/g. The other carotenes (Fig. 2A) and carotenoids had a high correlation between the two lines without significant mean differences. The total concentration of carotenoid and anthocyanins for the CIMMYT yellow lines converted to VMLs did not correlate (r = 0.029) and had no mean difference (*p*-value = 0.956). The ANOVA on the carotenoid profiling comparing CMLs and VMLs showed that only β-cryptoxanthin presented a significant difference (*p*-value = 0.04), with the VML group having a lower concentration (Fig. 2B).

**Table 1:** Carotenoids profile and total anthocyanin content of CMLs and VMLs

Sample	Lutein (µg/g)	Zeaxanthin (µg/g)	β-Cryptoxanthin (µg/g)	13-cis-BC (µg/g)	β-carotene (µg/g)	9-cis-BC (µg/g)	Total carotenoids (µg/g)	Total anthocyaninsµg/g)
CML 027	2.37 ± 0.06	5.01 ± 0.07	8.90 ± 0.08	0.56 ± 0.01	1.47 ± 0.03	0.48 ± 0.01	18.79 ± 0.17	0.0 ± 0.0
CML 305	0.85 ± 0.04	2.30 ± 0.06	8.92 ± 0.28	0.57 ± 0.01	1.46 ± 0.02	0.57 ± 0.01	14.67 ± 0.11	0.0 ± 0.0
CML 321	0.0 ± 0.0	0.25 ± 0.02	0.0 ± 0.0	0.0 ± 0.0	0.0 ± 0.0	0.0 ± 0.0	0.25 ± 0.0	0.0 ± 0.0
CML 327	2.57 ± 0.12	5.12 ± 0.06	2.47 ± 0.04	1.17 ± 0.07	3.71 ± 0.27	1.06 ± 0.21	16.10 ± 0.17	0.0 ± 0.0
CML 451	0.69 ± 0.03	1.65 ± 0.04	0.13 ± 0.01	0.22 ± 0.0	0.28 ± 0.0	0.23 ± 0.0	3.12 ± 0.05	0.0 ± 0.0
CML 490	0.0 ± 0.0	0.11 ± 0.0	0.0 ± 0.0	0.0 ± 0.0	0.0 ± 0.0	0.0 ± 0.0	0.10 ± 0.0	0.0 ± 0.0
CML 491	0.0 ± 0.0	0.12 ± 0.01	0.0 ± 0.0	0.0 ± 0.0	0.0 ± 0.0	0.0 ± 0.0	0.12 ± 0.0	0.0 ± 0.0
CML 492	0.0 ± 0.0	0.11 ± 0.0	0.0 ± 0.0	0.0 ± 0.0	0.0 ± 0.0	0.0 ± 0.0	0.11 ± 0.0	0.0 ± 0.0
CML 494	0.0 ± 0.0	0.13 ± 0.0	0.0 ± 0.0	0.0 ± 0.0	0.0 ± 0.0	0.0 ± 0.0	0.10 ± 0.0	0.0 ± 0.0
CML 496	1.22 ± 0.1	3.14 ± 0.20	4.59 ± 0.23	0.45 ± 0.03	0.84 ± 0.05	0.80 ± 0.01	11.04 ± 0.07	0.0 ± 0.0
VML 027	0.52 ± 0.06	2.04 ± 0.20	2.66 ± 0.08	0.55 ± 0.02	1.35 ± 0.03	0.65 ± 0.03	7.77 ± 0.05	300.6 ± 13.0
VML 305	2.11 ± 0.0	4.58 ± 0.47	4.90 ± 0.28	0.67 ± 0.04	1.62 ± 0.08	0.82 ± 0.01	14.7 ± 0.10	255.9 ± 16.8
VML 321	0.03 ± 0.1	0.18 ± 0.01	0.06 ± 0.0	0.18 ± 0.0	0.20 ± 0.20	0.19 ± 0.0	0.84 ± 0.01	133.5 ± 16.2
VML 327	2.91 ± 0.08	5.37 ± 0.11	3.45 ± 0.04	1.24 ± 0.06	3.55 ± 0.01	1.04 ± 0.02	17.56 ± 0.27	353.9 ± 20.9
VML 451	0.93 ± 0.03	2.65 ± 0.04	0.09 ± 0.0	0.25 ± 0.02	0.44 ± 0.05	0.31 ± 0.0	4.67 ± 0.03	335.7 ± 22.1
VML 490	0.0 ± 0.0	0.12 ± 0.0	0.0 ± 0.0	0.0 ± 0.01	0.00 ± 0.0	0.0 ± 0.0	0.12 ± 0.0	219.9 ± 38.1
VML 491	0.03 ± 0.01	0.14 ± 0.01	0.06 ± 0.0	0.19 ± 0.0	0.20 ± 0.01	0.19 ± 0.0	0.81 ± 0.0	262.8 ± 36.4
VML 492	0.04 ± 0.0	0.21 ± 0.0	0.08 ± 0.0	0.18 ± 0.0	0.18 ± 0.0	0.18 ± 0.0	0.87 ± 0.01	138.0 ± 17.2
VML 494	0.03 ± 0.0	0.15 ± 0.0	0.09 ± 0.0	0.19 ± 0.0	0.21 ± 0.0	0.19 ± 0.0	0.86 ± 0.01	300.6 ± 13.0
VML 496	0.11 ± 0.0	0.55 ± 0.0	0.49 ± 0.23	0.67 ± 0.0	1.76 ± 0.01	0.72 ± 0.01	4.30 ± 0.02	255.9 ± 16.8

*Mean values ± standard errors (σ/n^1/2^), n = 3. Carotenoids in fresh weight, anthocyanins in dry weight.

Carotenoid and anthocyanin concentrations are polygenic traits [8, 12], and their biosynthesis is species and tissue-dependent: A negative correlation between their total concentration was described for *Capsicum annum* and *Solanum tuberosum* [23, 24]. A genetically modified *Malus hupehensis* had an increase in transcripts for carotenoid biosynthesis but a decrease in the anthocyanin ones [22, 25]. For rose petals, no correlation between anthocyanin and carotenoid content was found [26, 27]. In maize kernels, carotenoids are produced in the vitreous and floury endosperm, and anthocyanins are synthesized in the aleurone layer, which could explain the observed lack of correlation [6, 7].

### Lysine and Protein Tryptophan Concentration in CMLs and VMLs

The concentration of tryptophan and protein lysine from CMLs and VMLs are displayed in Fig. 2C and D. The QPM lines stand out among the CIMMYT lines. Three of them were chosen: 490, 491, and 492 [28]. Both amino acid concentrations in VMLs were lower than in their parents. Therefore, the QPM trait was not preserved in the VMLs from a QPM origin. The VMLs 451 and 494 have a high concentration of just one of the two amino acids, which does not qualify for a QPM endosperm.

### Distinction Between CMLs and VMLs by DIMS

**Fig. 3:**
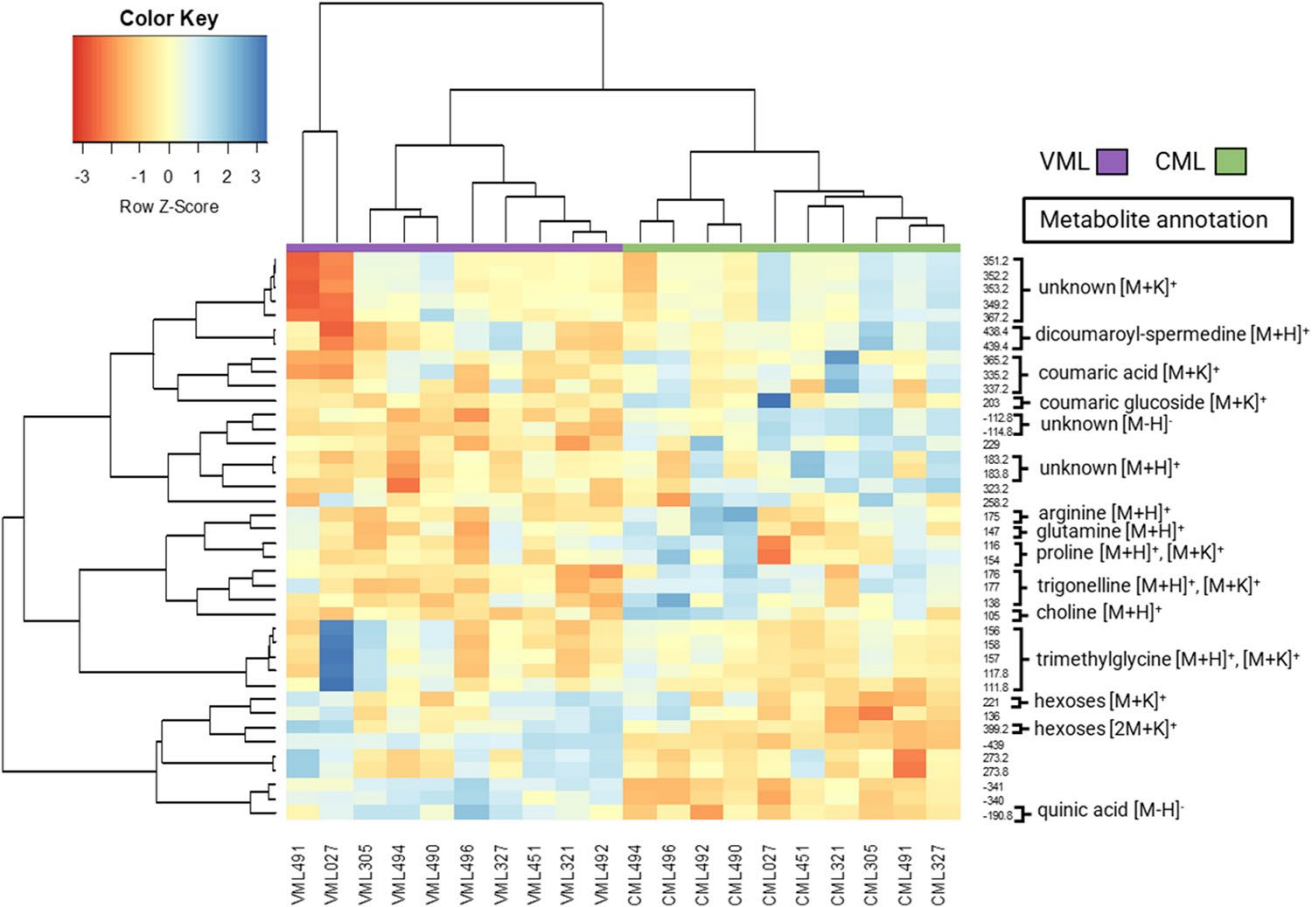
HCA for DIMS data from aqueous extraction of CMLs and VMLs. Blue represents a higher signal intensity, while red indicates a lower signal intensity. For the HCA, fifty ions with the lowest *p*-value obtained from an ANOVA model for the genotype factor (CML or VML) were chosen. CMLs and VMLs are separated into two clusters, indicating distinct metabolic identities

The whole-seed chemical profiles of VMLs and their parental CMLs were compared using different extraction media. The aqueous extraction separated the CMLs and VMLs into two clusters (Fig. 3). For the hierarchical cluster analysis (HCA), forty ions with the lowest *p*-value from the analysis of variance (ANOVA) were chosen, and several putative compounds were identified through fragmentation analyses (Fig. 3, [7, 20, 29, 30]). The annotated metabolites are: hexose ions at 221 [M + K]^+^ and 399 [2 M + K]^]+^ *m/z*, betaine ions such as trigonelline at 138 [M + H]^+^ and 176 [M + K]^+^ *m/z*, trimethylglycine at 118 [M + H]^+^ and 156 [M + K]^+^ *m/z,* and choline at 105 [M + H]^+^ *m/z.* The signals of amino acids such as proline are at 116 [M + H]^+^, glutamine is at 147 [M + H]^+^, and arginine is at 175 [M + H]^+^ *m/z*. Hexose ions are more intense in Vitamaize lines than in CMLs, and amino acid and betaine ions were more intense signals in the CMLs.

The DIMS fragmentation analysis of the methanolic extracts (Figures S12 and S13) indicated potassium adducts [M + K]^+^. Potassium adducts present little or no fragmentation, making their identification difficult [31]. The first cluster of 32 variables had a mass-to-charge ratio between 300 and 800, with higher signal intensities for the CMLs. Taking into account the extraction solvent and their high mass-to-charge values (> 600), they could be identified as glycerolipids [M + K]^+^. The most intense ions in the VML group belonged to malonic acid at 103 [M-H]^−^ *m/z* and hexoses at 217 [M + K-2H]^−^ *m/z*.

The intensities for 449 m*/z* and the MS/MS transition 449–287 for cyanidin 3-O-glycoside (449 g/mol) show that the anthocyanin with cyanidin base is abundant in the purple kernels (Figure S14A and B). The MS/MS transition of 519–271 corresponds to the pelargonidin malonylglucoside (519 g/mol) and is one of the most relevant compounds in the profile of the red VML 305 (Figure S14C and D).

The results indicate that the backcrossing for anthocyanin accumulation did not produce other metabolic modifications shared among all VMLs except for the anthocyanin trait. Three factors why backcrosses did not generate the expected changes were hypothesized:

1) Every VML has its unique set of genes introduced into the CML genome from the pigmented landrace (donor). Every introgressed gene in the genome of the CIMMYT lines has modified multiple routes in the kernel metabolism, not only those related to pigmentation. 2) The minimum impact on the whole seed metabolism by any metabolic modification in the aleurone monolayer. 3) A complex non-Mendelian inheritance is ruling on maize genetics [32]. A pigmented phenotype in the grain has a polygenic inheritance depending on several biosynthetic genes and the environment (cold temperature and UV radiation) to enhance anthocyanin production [8, 9, 21, 33]. In the backcrossing, the darker seeds were selected to continue the breeding, thus choosing other alleles that increase hexose and anthocyanin seed content at the same time.

The breeding by visual selection is also responsible for losing the QPM phenotype in anthocyanin-pigmented converted lines from CIMMYT QPM parent lines. Therefore, backcross breeding programs to introduce an anthocyanin-pigmented seed phenotype in a protein-quality endosperm (QPM) genetic background need molecular markers [11].

### Phylogeny of c1 and r1 Genes for the CMLs and VMLs

**Fig. 4:**
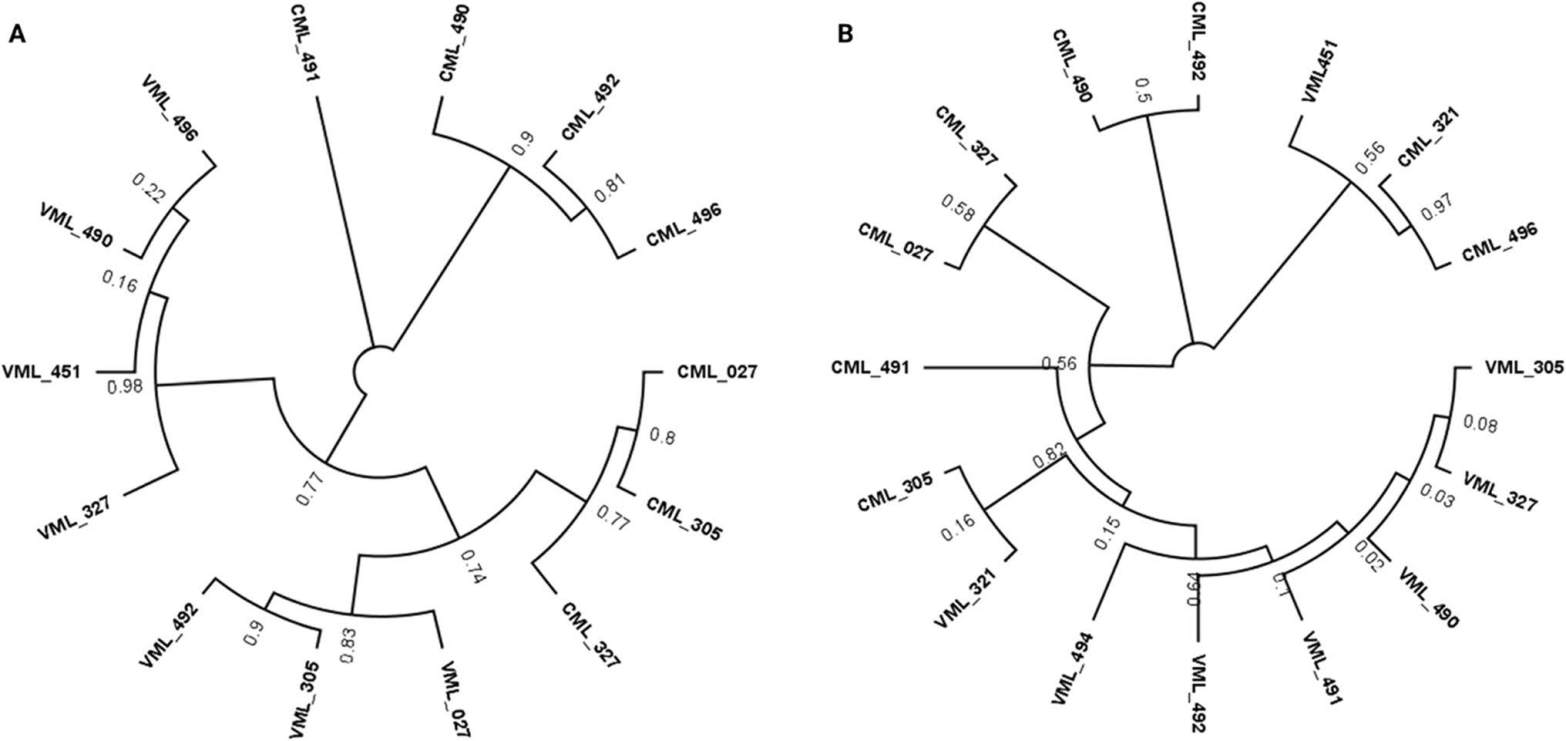
Maximum likelihood phylogenetic tree of the *c1* and *r1* gene sequences of the CMLs and VMLs. Phylogenetic position of members of the VML and CML, based on the *c1* (A) and *r1* (B) sequences. Only some of the VMLs have introgressed the two most relevant genes controlling the anthocyanin accumulation in the aleurone. The numbers at the nodes represent percentage levels of bootstrap support from 500 replications

The phylogenetic data for the *c1* (Fig. 4A) and *r1* (Fig. 4B) reveal that not all VMLs are closely related, contrary to what was expected from the original hypothesis, in which those gene sequences should come from the same pigmented donor parent. The results demonstrated that some VML sequences are phylogenetically closer to those of CMLs. In Fig. 4A, the phylogenetic tree shows two main branches, both subsequently bifurcating into two clades: one for VMLs and another for CMLs. The latter result implies that the donor parent used for the backcrossing was not homozygous in the *c1* gene.

In Fig. 4B, the phylogenetic tree showed that most VMLs were genetically related to each other at the *r1* gene, except for the VML 451, which was not in the VML clade. The *r1* gene is a complex gene that presents a non-Mendelian pattern inheritance. Further studies could test for heritable epigenetic silencing by a silenced allele (paramutation) that could explain why VML 451 had a similar DNA sequence to the non-pigmented CML [32].

## Conclusion

During the backcrossing for generating the Vitamaize lines, alleles of a donor parent with pigmented aleurone entered the genome of the CMLs. The overall metabolic modifications and the carotenoid profile were not consistent among all VMLs. Some VMLs had higher concentrations than their CML counterparts. Therefore, backcrossing for pigmentation produced individual changes to the seed metabolism depending on the introduced regions or *loci*.

Evaluating metabolic modifications in the aleurone is technically challenging since this tissue is only a monolayer. Thus, metabolic changes in the aleurone are difficult to detect in the whole seed. Choosing visually darker-pigmented grains also selected alleles that altered other metabolic routes related to the anthocyanin content. In addition, visual selection resulted in the loss of the QPM trait, a nutritionally desirable and genetically complex phenotype.

Therefore, any future work regarding maize improvement for a nutraceutical target, such as high anthocyanin content, must include molecular and biochemical analyses to confirm traits such as a QPM endosperm after each cross.

### Supplementary Information

Below is the link to the electronic supplementary material.

Supplementary file1 (DOCX 27 KB)

Supplementary file2 (PPTX 27250 KB)

## Data Availability

Mass spectrometry data are available from Zenodo: Peniche-Pavía, H. A., & González-Rodríguez, T. (2023). CMLs vs VMLs DIMS [Data set]. 10.5281/zenodo.8423433.
