# Supplementary material for: Backcrossing Modulates the Metabolic Profiles of Anthocyanin-Pigmented ‘Vitamaize’ Lines Derived from Elite Maize Lines
Source: Plant Foods Hum Nutr. 2024 Feb 9;79(1):202–8. doi: 10.1007/s11130-024-01155-0 (PMC10891256; doi:10.1007/s11130-024-01155-0)
Supplement: Supplementary file 1 — Supplementary file1 (DOCX 27 KB) [file 11130_2024_1155_MOESM1_ESM.docx]

**Supplementary Material 1**

**MATERIALS AND METHODS**

**Backcrossing Modulates the Metabolic Profiles of Anthocyanin-Pigmented ‘Vitamaize’ Lines Derived from Elite Maize Lines**

Héctor Arturo Peniche-Pavía^1^, Tzitziki González-Rodríguez^1,2^, Axel Tiessen^1,†^, Silvero García-Lara^2^ and Robert Winkler^1,*^

^1^CINVESTAV Unidad Irapuato and UGA-Langebio Irapuato, Km. 9.6 Libramiento Norte Carr. Irapuato-León, PC 36824 Irapuato, Gto., Mexico.

^2^Tecnológico de Monterrey, School of Engineering and Sciences, EIC, Ave. Eugenio Garza Sada 2501, PC 64849 Monterrey, N.L, Mexico

We dedicate this article to Prof. Axel Tiessen Favier († 2020), who created the Vitamaize lines.

# *Corresponding author: [robert.winkler@cinvestav.mx](mailto:robert.winkler@cinvestav.mx)

**Materials and Methods**

Sample collection

All the samples of CIMMYT maize lines (CMLs) and Vitamaize lines (VMLs) were grown under optimal agronomic conditions for subtropical conditions and were self-pollinated [1]. They were cultivated and maintained in optimal greenhouse conditions from March to August 2018 in Irapuato, Mexico (N 20.71933, W-101.33088; 1720 meters above mean sea level, MAMSL). We harvested four ears from each maize line. The maize line selection included the same amount of CMLs with white endosperm (CML 321, CML 490, CML 491, CML 492, and CML 494) and with yellow endosperm (CML 027, CML 305, CML 327, CML 451 and CML 496), and their respective counterpart VMLs.

Sample preparation

We selected ten random seeds in the R6 development stage and ground them in a ball milling using a Retsch Mill MM 340 (Retsch, Haan, Germany). Subsequently, we weighed 10 mg in a two mL microtube for each extraction (water, MeOH with 0.1% FA, and MeOH with 1% TFA). The water and methanol were LC-MS grades water (TEDIA, Fairfield, USA). Formic acid (FA) and Trifluoroacetic acid (TFA) were Optima™ LC-MS grade (Fisher Chemical, Leicestershire, UK).

All samples were extracted in an ultrasound bath for 10 minutes at room temperature (T ≈ 25 °C). Finally, the samples were centrifuged at 12,000 g in a 5415R centrifuge (Eppendorf, Hamburg, Germany) and filtered with Target® PTFE filters (pore size 0.2 μm, 30 mm). The injection flow was adjusted to 10 µl/min with a Fisherbrand ™ infusion pump (Waltham, MA, USA).

Direct infusion mass spectrometry (DIMS)

The equipment used for all mass spectrometry experiments, including fragmentation, was Waters' TQD (Milford, MA, USA). The parameters were capillary voltage at 3 kV, 30 V for the cone, the source temperature at 150 °C, and the desolvation gas at 250 °C. The desolvation gas flow was 250 l/h, and the flow in the cone was 50 l/h. The acquisition mode was in MCA from -20 to -600 *m/z* for negative and 20 to 1500 *m/z* for positive ion modes. Each sample reading lasted ≈5 min and included six technical replicas (0.3 minutes for each scan) in both ionization modes. We measured four biological replicates for each maize line.

We used the MassLynx™ 4.1 software (Waters, Milford, MA, USA) for all DIMS experiments. The Hierarchical Clustering Analysis (HCA) used ions (variables) with the lowest p-value or the highest mean decrease accuracy. In the fragmentation method, we selected three voltages for the CID: 10 V, 20 V, and 30 V. The MS/MS protocol to compare the relative acylated maize anthocyanins content is described in Magaña-Cerino et al. (2020).

Statistical analysis and mass spectrometry data processing

All MS data were preprocessed and analyzed in the R environment [3]. For the preprocessing and its analysis, we used the packages "ChemometricsWithR" [4] and "Agricolae" [5], as described in [6]. We included a supervised classification algorithm, Random forest (RF), to evaluate which ions (variables) were necessary to classify the samples as CML or VML. We implemented the algorithm within the R package "randomForest" [7]. The RF classifies the samples according to a sample label and ranks the importance of the variables (ions) through the mean decrease accuracy value for the classification between CML and VML. Then, we plotted the HCA's 50 top ions with the highest mean decrease accuracy value.

The total content of monomeric anthocyanins by spectrophotometry

The differential pH method in a spectrophotometer was employed as described by Lee et al. (2005) with a minor modification. The protocol measured an extract of the sample in two solutions: a buffer at pH = 1 (25 mM NaCl, adjusted with HCl) and a pH = 4.5 (0.4 M sodium acetate, adjusted with HCl). 20 mg of maize powder was weighed in two microtubes and then extracted twice to obtain the total anthocyanin content. After adding the solvent, it was mixed in a vortex mixer and left for 90 min under constant stirring in a ThermoMixer® at four °C. After incubation, extracts were centrifuged for ten minutes at 12,000 g. Then, the supernatant was recovered to measure at two lengths of 520 and 700 nm. Each sample had biological triplicates. The calculation for the anthocyanin concentration was as follows:

**Anthocyanin total content** (the equivalent of cyanidin 3-*O*-glucoside in μg/g):

$$\frac{\Delta AxMWxV}{\varepsilon xlxW}$$

Where *ΔA* = (A_520nm_-A_700nm_) _pH1_- (A_520nm_-A_700nm_) _pH 4.5_; *MW* (Molecular Weight) = 449.2 g/mol for cya-3-*O*-glc; *V* is for volume = 0.0013 l; *l* is distance, in cm; *ε* (molar attenuation coefficient) = 26 900 l x mol^–1^ x cm^–1^; *W* is for the sample dried weight (0.02 g).

Carotenoid Profiling by HPLC

This analysis followed the CIMMYT protocol [9] and was conducted at their facilities (Texcoco, Mexico). The process involved extracting and hydrolyzing esterified maize carotenoids and evaluating the free carotenoids using HPLC.

Quantification of tryptophan and lysine by spectrophotometry

Both quantifications were performed at CIMMYT using the methodology described by Galicia et al. (2009). The protocols include a protein hydrolysis process before the amino acid quantification. The targeted amino acids undergo specific reactions: lysine reacts with 2-chloro-3,5-dinitropyridine, and tryptophan reacts with glyoxylic acid under acidic conditions.

Primers design

We employed the Primer-BLAST software (NCBI-NIH) for the primer design for the genes ZmMYB1 (*c1*, GRMZM2G005066) and ZmbHLH1 (*r1*, GRMZM5G822829). The parameters for the oligonucleotide design were amplicon length (800-1200), Tm of 56 to 64 ° C, and a Tm difference of 3. The accessions used for the multiple sequence alignment include the *C1*, *C1-I*, and *c1-B73* alleles available from the NCBI database. The design of primers for the *c1* gene included exon 1 to exon 3. The accessions for the *C1* allele were M37153.1, X06333.1, AF320614.3, and AF320613.3; for the *C1-I*, we included the accession X52201. 1, and *c1-B73* (NC_050104.1: c11118364-11117170).

For the primers of the *r1* gene, all the available alleles include at least the first exon, which is present in the transcript expressed in the aleurone [10]. These alleles are R-d: Catspaw (U93178.1), alc1 (DQ414252.1), r-st (AF380388.1), and r1-Lcm1 (AF135456) and r1-B73 (NC_050105.1: 141187431-141196825). We used the Beacon Designer program (Primer Biosoft) to confirm the calculated Tm, absence of the dimerizations, and secondary structures. The sequence of the primers for the *c1* gene are C1_E13A_Fw (upstream primer 5′- GTGGACGAGCAAGGAGGAC-3′) and C1_E13A_Rv (downstream primer 5′- CTGACA-GCGGAGCCAGTC-3′); for the *r1* gene: R1_PIEA_Fw (upstream primer 5′- CGCCCT-CTTCTGGTCCATTT-3′) and R1_PIEB_Rv (downstream primer 5′- GACCGTACCGATGTTCTCGT-3 ′). The size of the amplified products was 989 bp for the C1_E13A pair and 1086 bp for the R1_PIEA pair. The synthesis of the primers was carried out in the company T4Oligo® (Irapuato, Mexico).

Plant material for DNA extraction

The maize leaves were harvested in the V3 development stage, collected directly into liquid nitrogen, and kept at -80 ° C until processing. The collected tissues were ground in a mortar with a pestle and liquid nitrogen. Then, 200 to 400 mg of frozen tissue was weighed into a 2 mL microtube.

DNA extraction protocol

A CTAB buffer extraction was used [11]. In this protocol, 1 mL of CTAB buffer (1% CTAB, 100 mM Tris-7.5, 700 mM NaCl, 50 mM EDTA) was added to each 2 ml microtube with plant tissue and incubated for 90 min at 65 °C in a ThermoMixer® (Eppendorf), with a constant movement at 400 rpm. Subsequently, the samples were mixed for 10 min with 1 mL of CHCl_3_/octanol (24: 1) and centrifuged. Then, the 700 µl of the aqueous phase was removed and incubated with 10 µl RNase for 30 min; the DNA was precipitated with isopropanol and washed with ethanol. It ends by drying at room temperature. A spectrometric analysis determined the DNA quality and quantity, followed by an agarose gel electrophoresis to check the DNA integrity.

Polymerase chain reaction

The amplification conditions consisted of an initial denaturation cycle at 98 °C for 3 min, followed by 30 cycles of 95 °C for 30 s, 60 °C for 30 s, 72 °C for 30 s, as well as the final elongation step of 72 °C for 5 min. The PCR products were purified using the commercial PureLink ™ Quick PCR package (Invitrogen; Vilna, LT). The amplicons were sequenced in the National Laboratory of Genomics for Biodiversity (LANGEBIO), CINVESTAV- Advanced Genomics Unit.

Phylogenetic analysis

The phylogenetic trees were performed using the Maximum likelihood method of MEGA version 11 [12], according to the nucleotide sequence with 500 Bootstrap replicas. The phylogenetic trees from MEGA were exported to the software FigTree v1.4.4. [13] to optimize their visualization of them.

# **References**

1. Tiessen-Favier A, Escalante-Aburto A, Espinosa-Leal C, García-Lara S (2022) Novel Combination of the Biophysical, Nutritional, and Nutraceutical Properties in Subtropical Pigmented Maize Hybrids. Plants 11:. https://doi.org/10.3390/plants11233221

2. Magaña-Cerino JM, Tiessen A, Soto-Luna IC, et al (2020) Consumption of nixtamal from a new variety of hybrid blue maize ameliorates liver oxidative stress and inflammation in a high-fat diet rat model. J Funct Foods 72:104075. https://doi.org/10.1016/j.jff.2020.104075

3. R Core Team (2022) R: A Language and Environment for Statistical Computing

4. Wehrens R (2011) Chemometrics With R: Multivariate Data Analysis in the Natural Sciences and Life Sciences. Springer Berlin Heidelberg, Berlin, Heidelberg

5. de Mendiburu F (2019) Agricolae: Statistical Procedures for Agricultural Research. In: R Packag. version 1.3-1. https://cran.r-project.org/package=agricolae. Accessed 20 Mar 2020

6. Peniche-Pavía HA, Tiessen A (2020) Anthocyanin Profiling of Maize Grains Using DIESI-MSQD Reveals That Cyanidin-Based Derivatives Predominate in Purple Corn, whereas Pelargonidin-Based Molecules Occur in Red-Pink Varieties from Mexico. J Agric Food Chem 68:5980–5994. https://doi.org/10.1021/acs.jafc.9b06336

7. Liaw A, Wiener M (2002) Classification and Regression by randomForest. R news 2:18–22

8. Lee J, Durst RW, Wrolstad RE (2005) Determination of total monomeric anthocyanin pigment content of fruit juices, beverages, natural colorants, and wines by the pH differential method: Collaborative study. J AOAC Int 88:1269–1278. https://doi.org/10.1093/jaoac/88.5.1269

9. Galicia L, Nurit E, Rosales A, Palacios–Rojas N (2009) Laboratory protocols 2009: Maize nutrition quality and Plant tissue analysis laboratory. CIMMYT, Mexico City

10. Procissi A, Piazza P, Tonelli C (2002) A maize r1 gene is regulated post-transcriptionally by differential splicing of its leader. Plant Mol Biol 49:239–248. https://doi.org/10.1023/A:1014959230492

11. CIMMYT (2005) Laboratory Protocols: CIMMYT Applied Molecular Genetics Laboratory, Third. CIMMYT, Mexico City

12. Tamura K, Stecher G, Kumar S (2021) MEGA11: Molecular Evolutionary Genetics Analysis Version 11. Mol Biol Evol 38:3022–3027. https://doi.org/10.1093/molbev/msab120

13. Rambaut A (2014) FigTree software v. 1.4.4
