# Supplementary material for: Backcrossing Modulates the Metabolic Profiles of Anthocyanin-Pigmented ‘Vitamaize’ Lines Derived from Elite Maize Lines
Source: Plant Foods Hum Nutr. 2024 Feb 9;79(1):202–8. doi: 10.1007/s11130-024-01155-0 (PMC10891256; doi:10.1007/s11130-024-01155-0)
Supplement: Supplementary file 2 — Supplementary file2 (PPTX 27250 KB) [file 11130_2024_1155_MOESM2_ESM.pptx]

## Slide 1
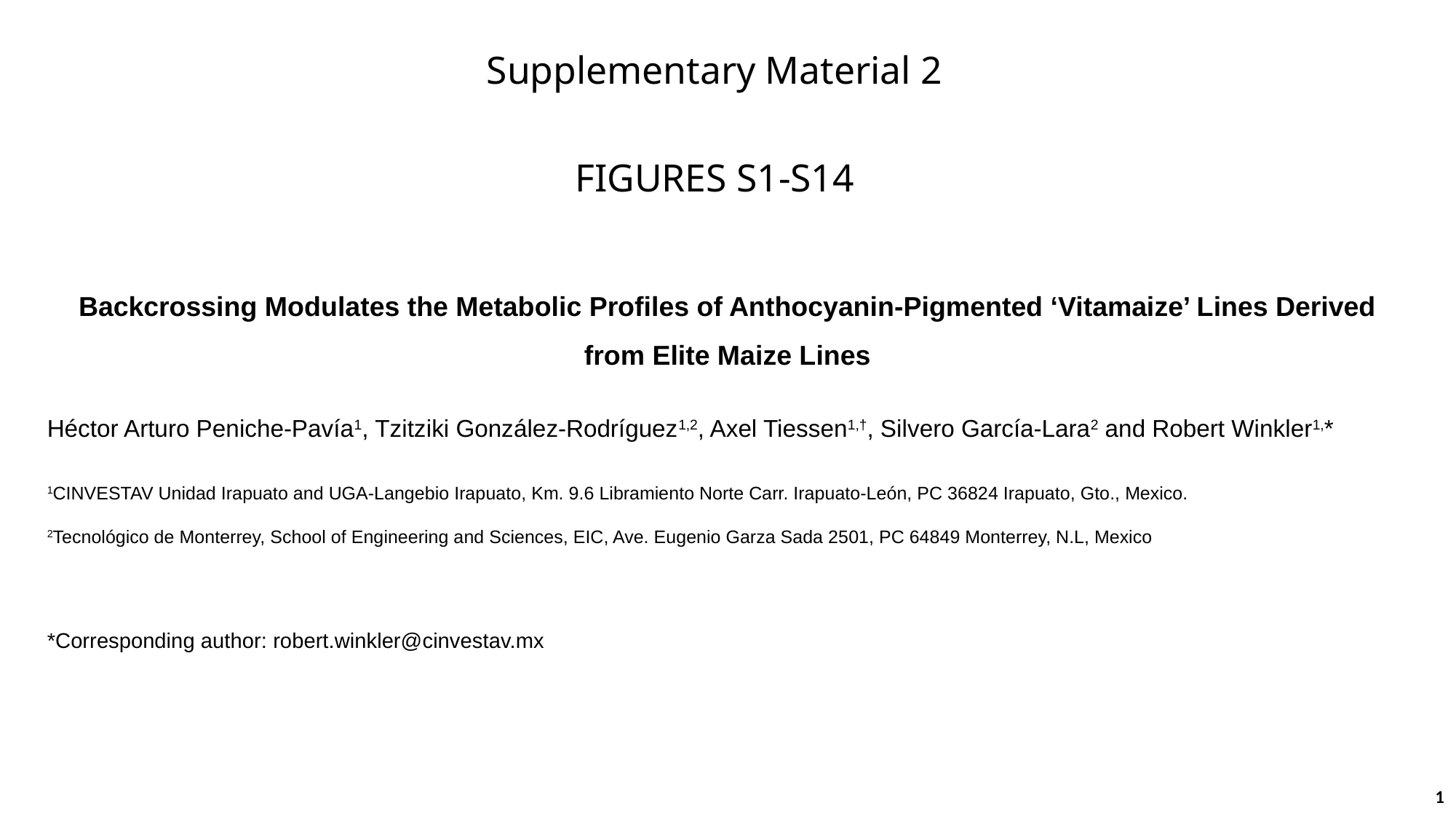

Supplementary Material 2
FIGURES S1-S14
Backcrossing Modulates the Metabolic Profiles of Anthocyanin-Pigmented ‘Vitamaize’ Lines Derived from Elite Maize Lines
Héctor Arturo Peniche-Pavía1, Tzitziki González-Rodríguez1,2, Axel Tiessen1,†, Silvero García-Lara2 and Robert Winkler1,*
1CINVESTAV Unidad Irapuato and UGA-Langebio Irapuato, Km. 9.6 Libramiento Norte Carr. Irapuato-León, PC 36824 Irapuato, Gto., Mexico.
2Tecnológico de Monterrey, School of Engineering and Sciences, EIC, Ave. Eugenio Garza Sada 2501, PC 64849 Monterrey, N.L, Mexico
*Corresponding author: robert.winkler@cinvestav.mx
1

## Slide 2
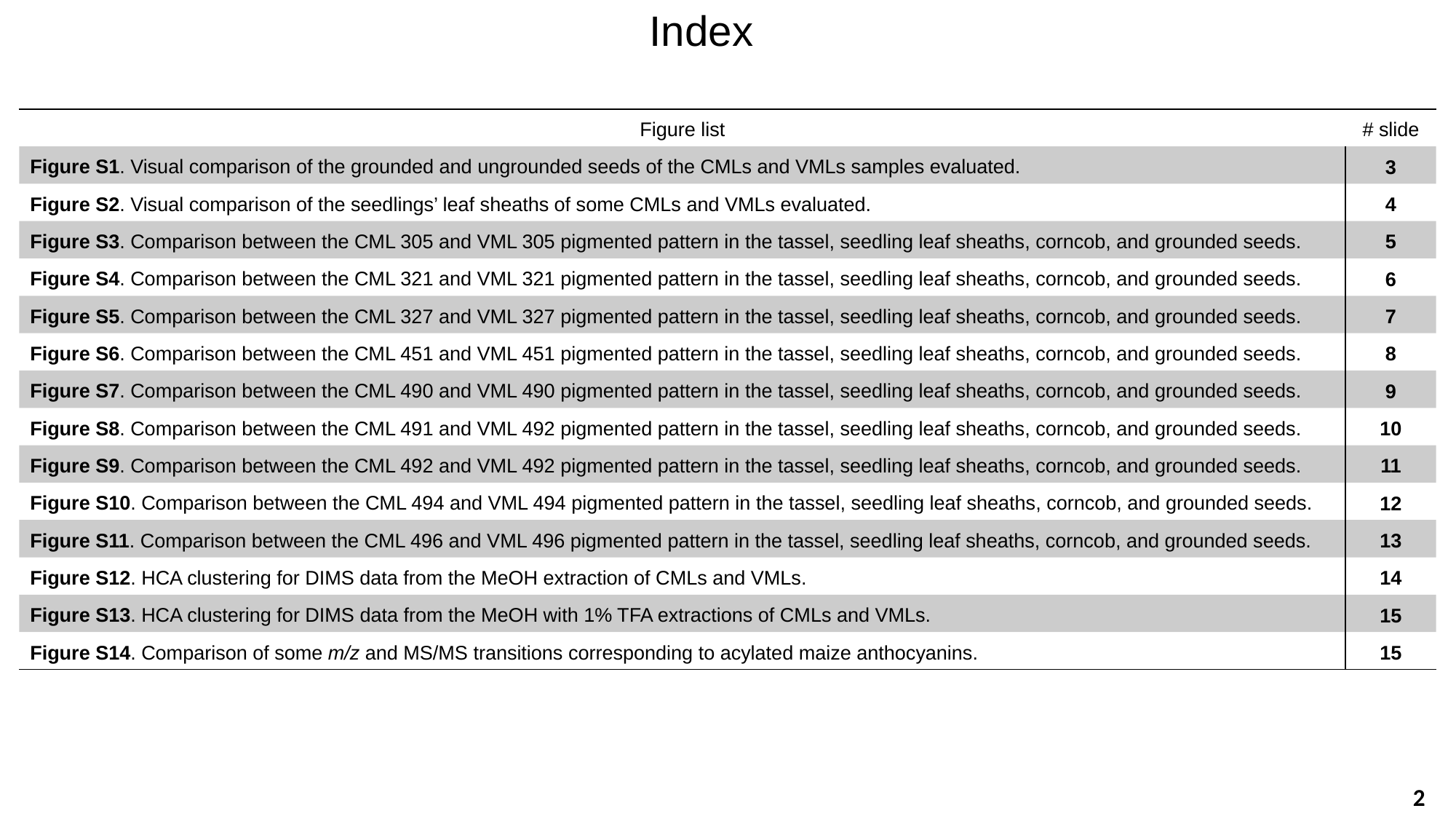

# Index
| Figure list | # slide |
| --- | --- |
| Figure S1. Visual comparison of the grounded and ungrounded seeds of the CMLs and VMLs samples evaluated. | 3 |
| Figure S2. Visual comparison of the seedlings’ leaf sheaths of some CMLs and VMLs evaluated. | 4 |
| Figure S3. Comparison between the CML 305 and VML 305 pigmented pattern in the tassel, seedling leaf sheaths, corncob, and grounded seeds. | 5 |
| Figure S4. Comparison between the CML 321 and VML 321 pigmented pattern in the tassel, seedling leaf sheaths, corncob, and grounded seeds. | 6 |
| Figure S5. Comparison between the CML 327 and VML 327 pigmented pattern in the tassel, seedling leaf sheaths, corncob, and grounded seeds. | 7 |
| Figure S6. Comparison between the CML 451 and VML 451 pigmented pattern in the tassel, seedling leaf sheaths, corncob, and grounded seeds. | 8 |
| Figure S7. Comparison between the CML 490 and VML 490 pigmented pattern in the tassel, seedling leaf sheaths, corncob, and grounded seeds. | 9 |
| Figure S8. Comparison between the CML 491 and VML 492 pigmented pattern in the tassel, seedling leaf sheaths, corncob, and grounded seeds. | 10 |
| Figure S9. Comparison between the CML 492 and VML 492 pigmented pattern in the tassel, seedling leaf sheaths, corncob, and grounded seeds. | 11 |
| Figure S10. Comparison between the CML 494 and VML 494 pigmented pattern in the tassel, seedling leaf sheaths, corncob, and grounded seeds. | 12 |
| Figure S11. Comparison between the CML 496 and VML 496 pigmented pattern in the tassel, seedling leaf sheaths, corncob, and grounded seeds. | 13 |
| Figure S12. HCA clustering for DIMS data from the MeOH extraction of CMLs and VMLs. | 14 |
| Figure S13. HCA clustering for DIMS data from the MeOH with 1% TFA extractions of CMLs and VMLs. | 15 |
| Figure S14. Comparison of some m/z and MS/MS transitions corresponding to acylated maize anthocyanins. | 15 |
2

## Slide 3
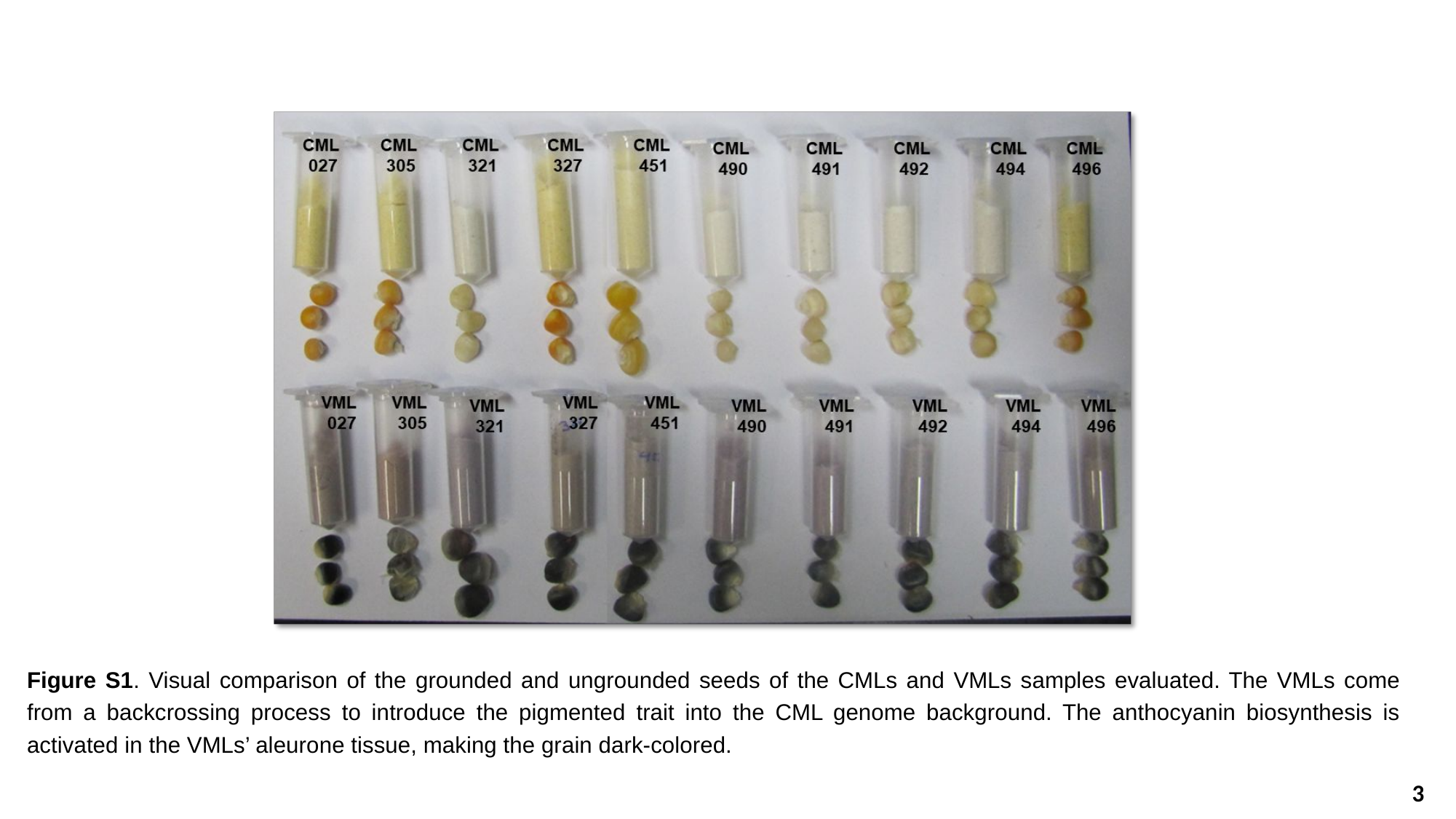

Figure S1. Visual comparison of the grounded and ungrounded seeds of the CMLs and VMLs samples evaluated. The VMLs come from a backcrossing process to introduce the pigmented trait into the CML genome background. The anthocyanin biosynthesis is activated in the VMLs’ aleurone tissue, making the grain dark-colored.
2

## Slide 4
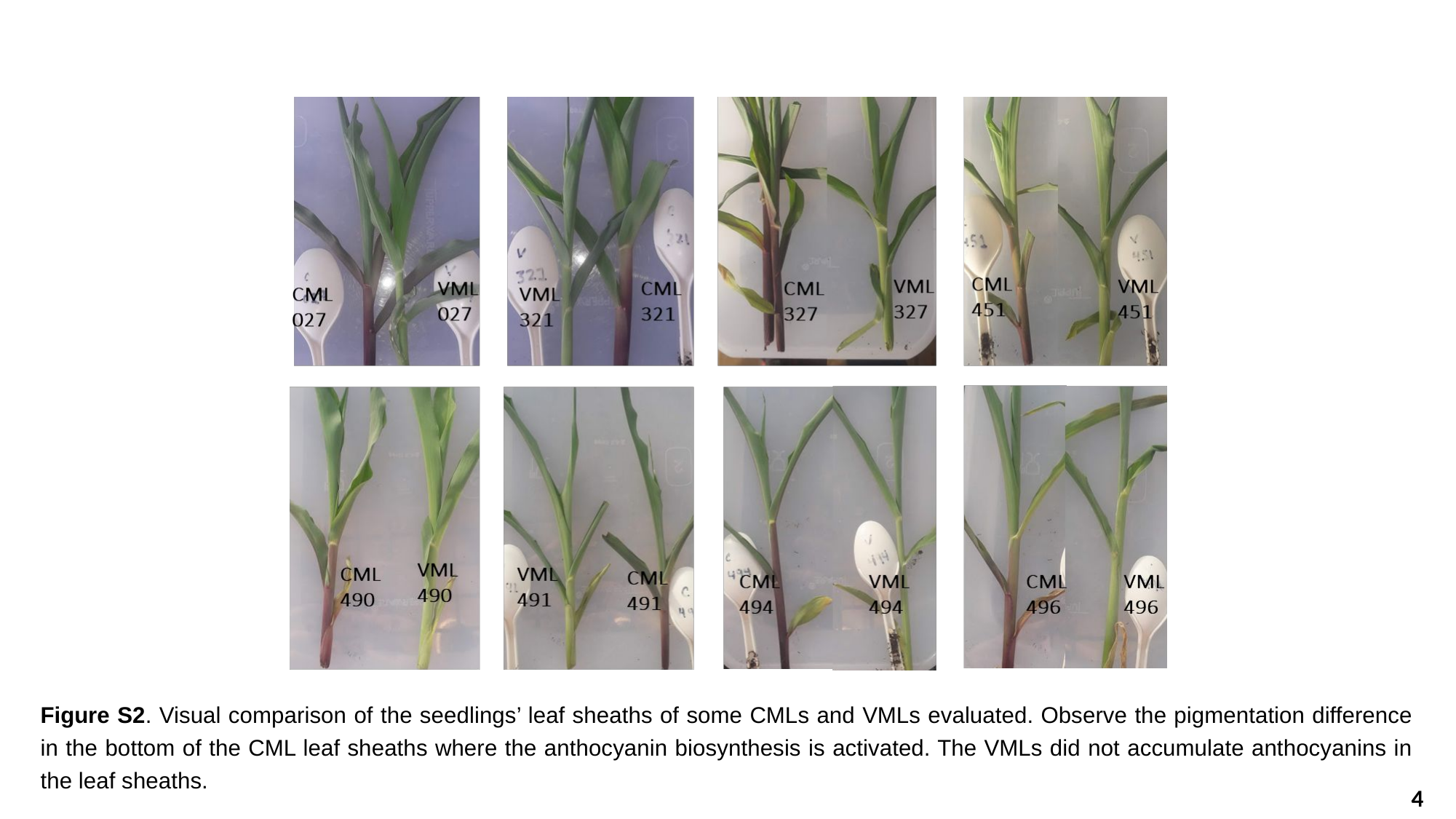

Figure S2. Visual comparison of the seedlings’ leaf sheaths of some CMLs and VMLs evaluated. Observe the pigmentation difference in the bottom of the CML leaf sheaths where the anthocyanin biosynthesis is activated. The VMLs did not accumulate anthocyanins in the leaf sheaths.
2

## Slide 5
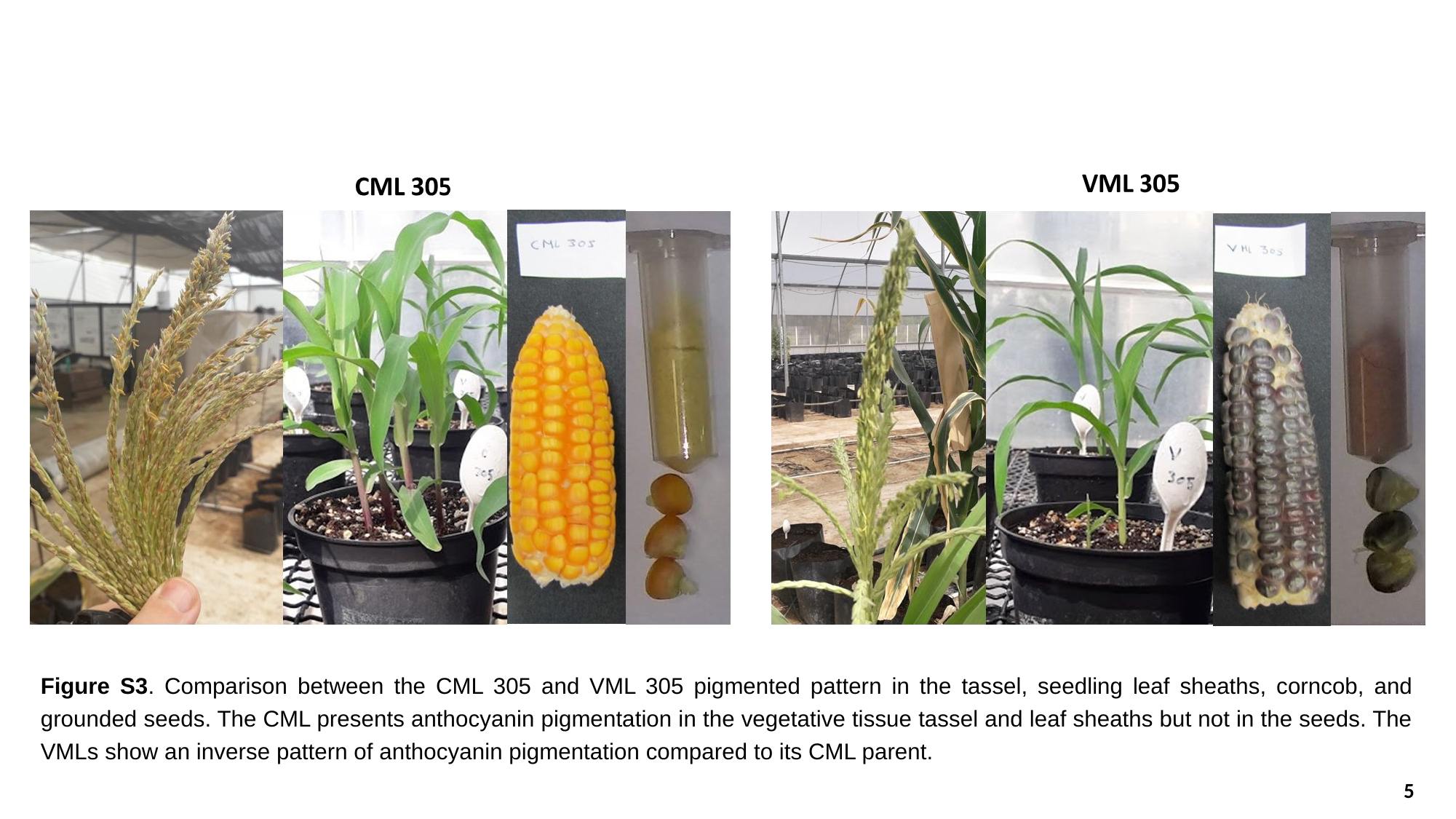

Figure S3. Comparison between the CML 305 and VML 305 pigmented pattern in the tassel, seedling leaf sheaths, corncob, and grounded seeds. The CML presents anthocyanin pigmentation in the vegetative tissue tassel and leaf sheaths but not in the seeds. The VMLs show an inverse pattern of anthocyanin pigmentation compared to its CML parent.
2

## Slide 6
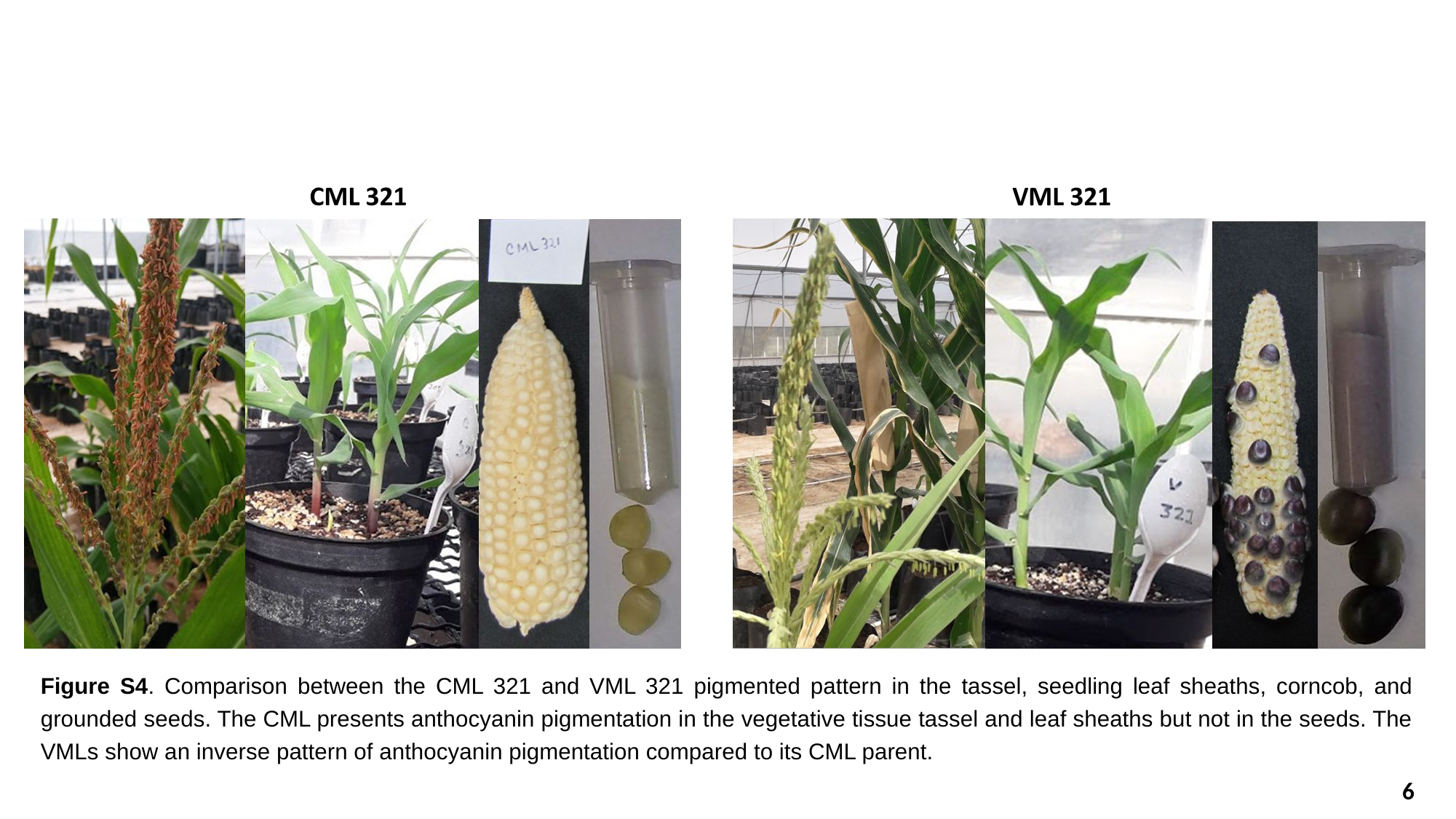

Figure S4. Comparison between the CML 321 and VML 321 pigmented pattern in the tassel, seedling leaf sheaths, corncob, and grounded seeds. The CML presents anthocyanin pigmentation in the vegetative tissue tassel and leaf sheaths but not in the seeds. The VMLs show an inverse pattern of anthocyanin pigmentation compared to its CML parent.
2

## Slide 7
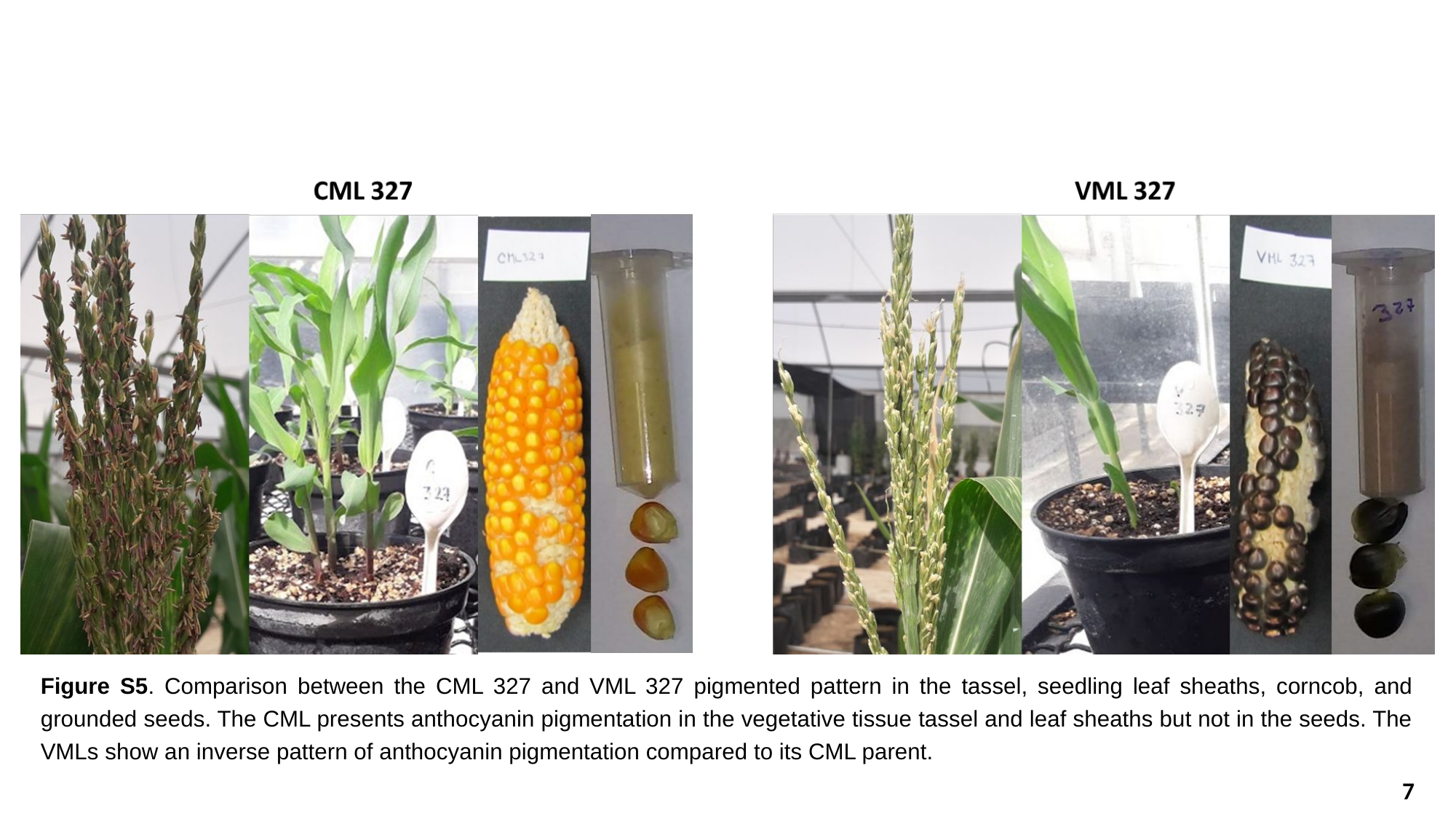

Figure S5. Comparison between the CML 327 and VML 327 pigmented pattern in the tassel, seedling leaf sheaths, corncob, and grounded seeds. The CML presents anthocyanin pigmentation in the vegetative tissue tassel and leaf sheaths but not in the seeds. The VMLs show an inverse pattern of anthocyanin pigmentation compared to its CML parent.
2

## Slide 8
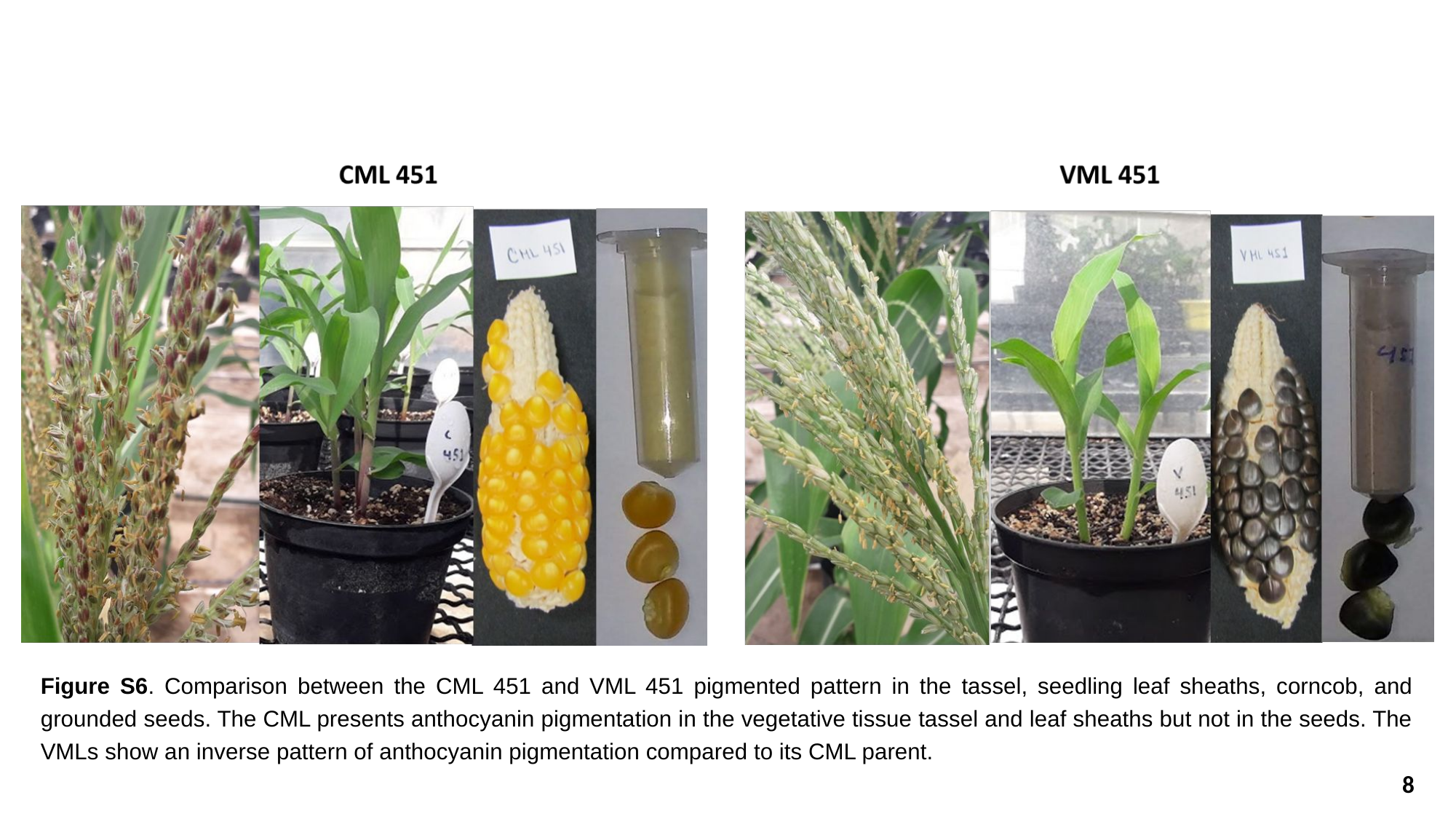

Figure S6. Comparison between the CML 451 and VML 451 pigmented pattern in the tassel, seedling leaf sheaths, corncob, and grounded seeds. The CML presents anthocyanin pigmentation in the vegetative tissue tassel and leaf sheaths but not in the seeds. The VMLs show an inverse pattern of anthocyanin pigmentation compared to its CML parent.
2

## Slide 9
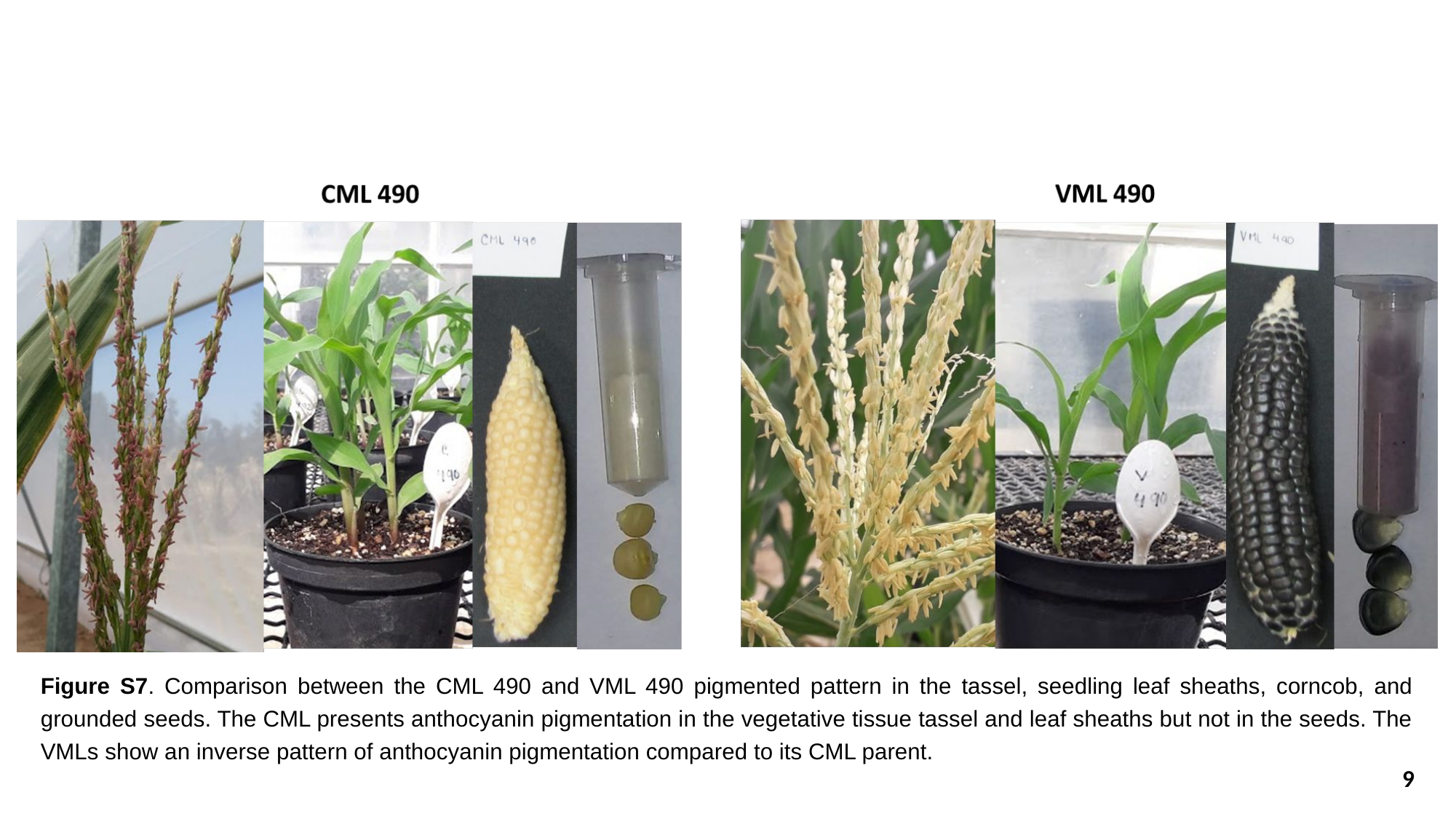

Figure S7. Comparison between the CML 490 and VML 490 pigmented pattern in the tassel, seedling leaf sheaths, corncob, and grounded seeds. The CML presents anthocyanin pigmentation in the vegetative tissue tassel and leaf sheaths but not in the seeds. The VMLs show an inverse pattern of anthocyanin pigmentation compared to its CML parent.
2

## Slide 10
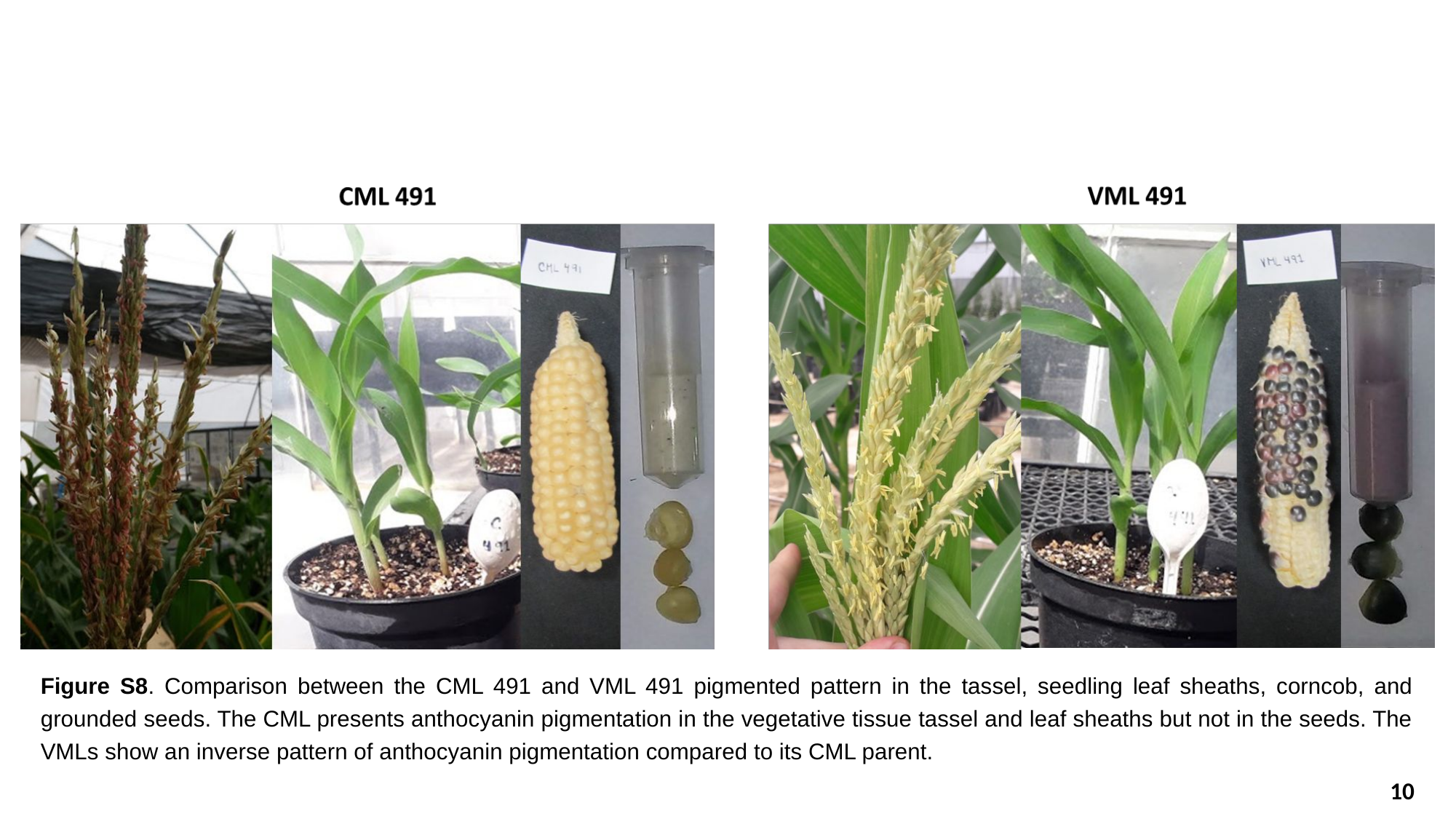

Figure S8. Comparison between the CML 491 and VML 491 pigmented pattern in the tassel, seedling leaf sheaths, corncob, and grounded seeds. The CML presents anthocyanin pigmentation in the vegetative tissue tassel and leaf sheaths but not in the seeds. The VMLs show an inverse pattern of anthocyanin pigmentation compared to its CML parent.
2

## Slide 11
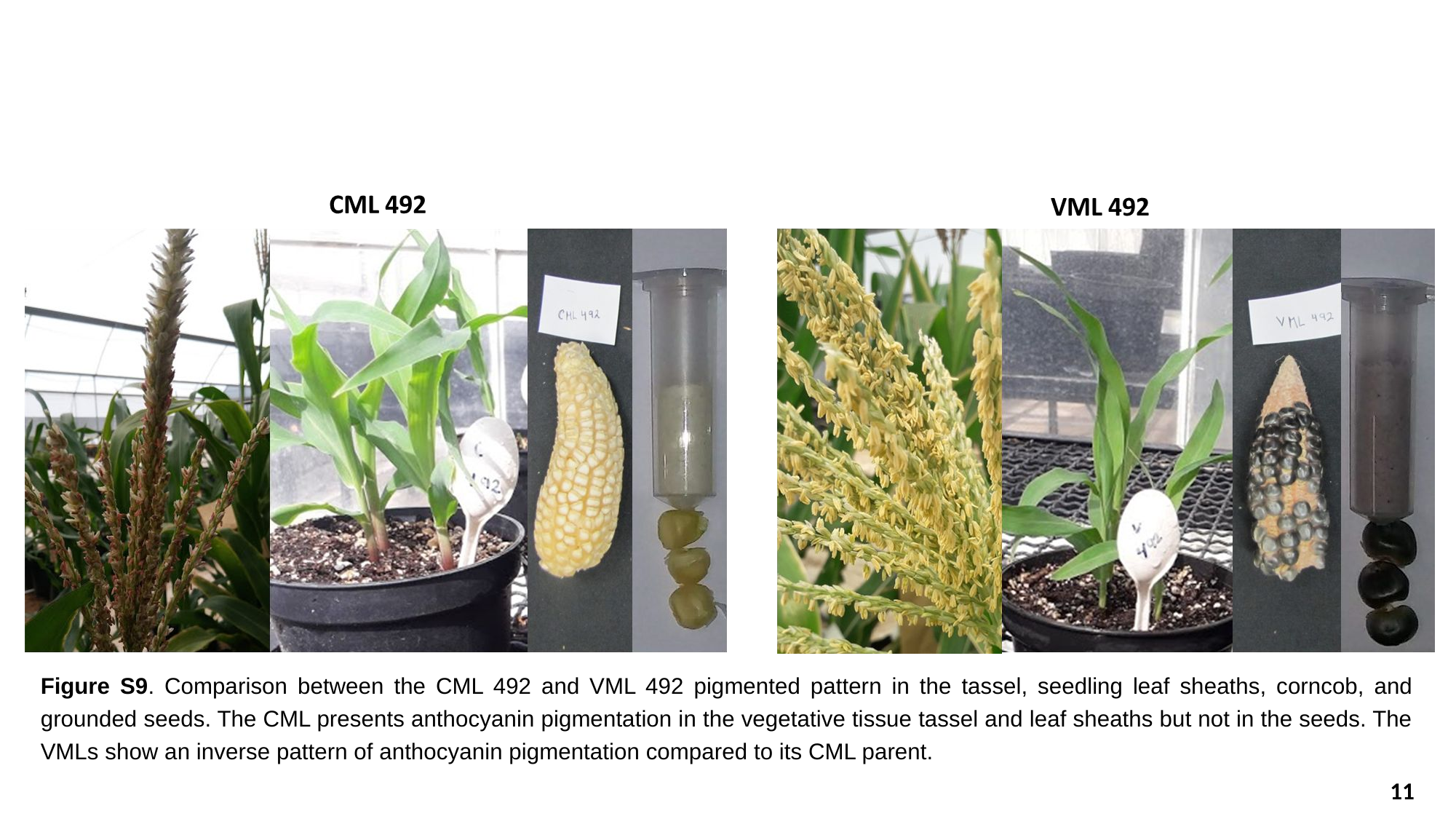

Figure S9. Comparison between the CML 492 and VML 492 pigmented pattern in the tassel, seedling leaf sheaths, corncob, and grounded seeds. The CML presents anthocyanin pigmentation in the vegetative tissue tassel and leaf sheaths but not in the seeds. The VMLs show an inverse pattern of anthocyanin pigmentation compared to its CML parent.
2

## Slide 12
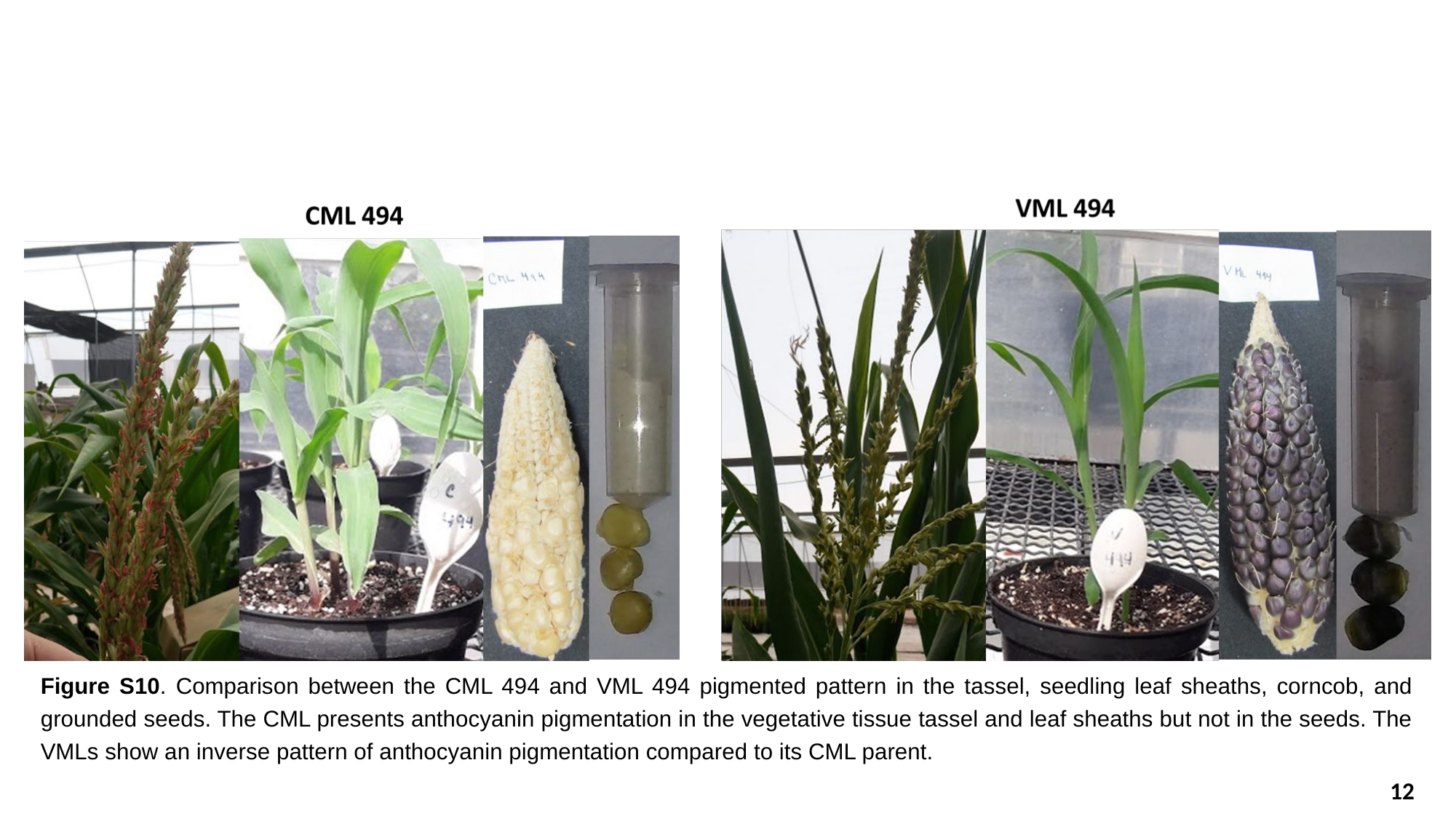

Figure S10. Comparison between the CML 494 and VML 494 pigmented pattern in the tassel, seedling leaf sheaths, corncob, and grounded seeds. The CML presents anthocyanin pigmentation in the vegetative tissue tassel and leaf sheaths but not in the seeds. The VMLs show an inverse pattern of anthocyanin pigmentation compared to its CML parent.
2

## Slide 13
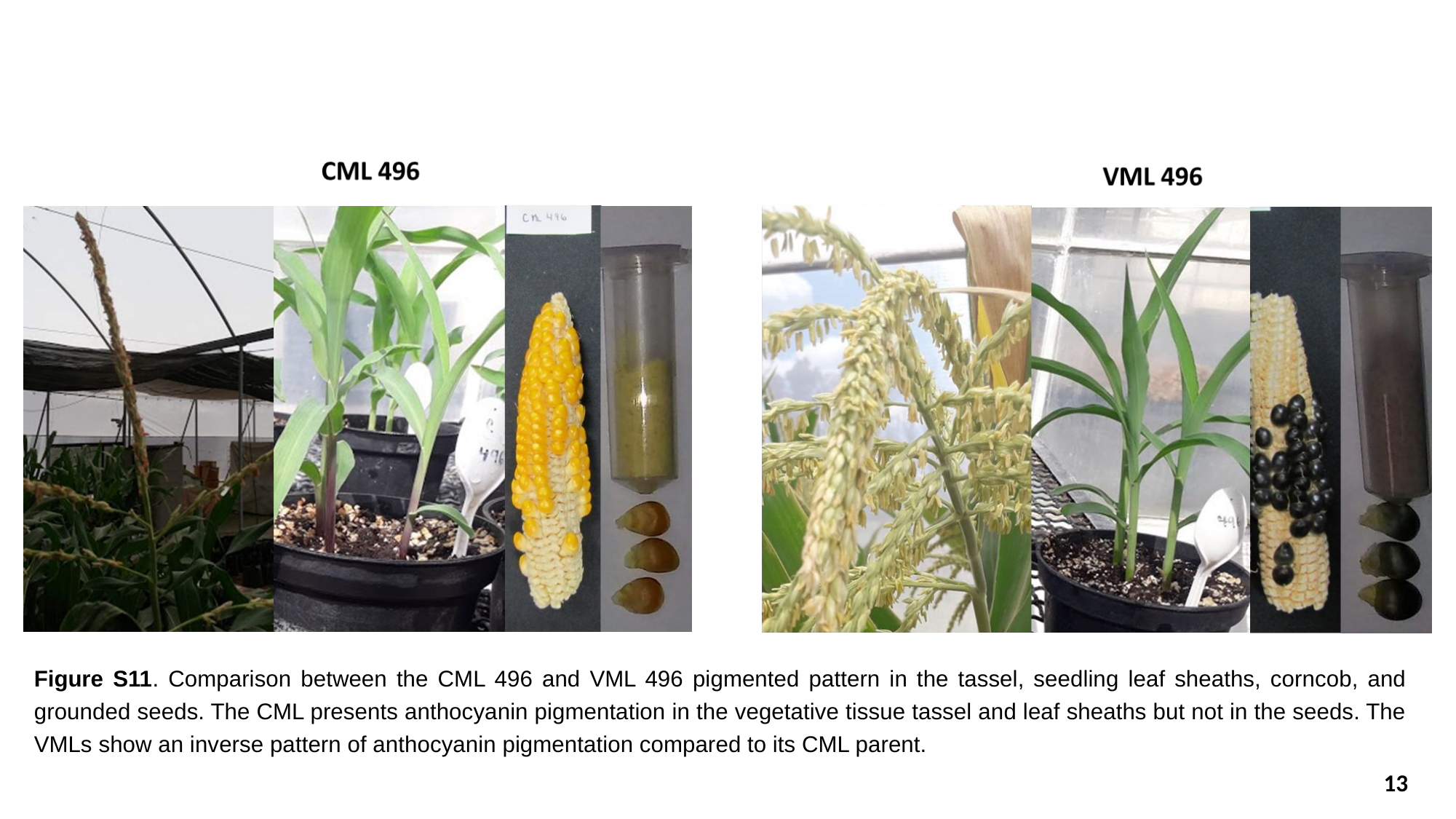

Figure S11. Comparison between the CML 496 and VML 496 pigmented pattern in the tassel, seedling leaf sheaths, corncob, and grounded seeds. The CML presents anthocyanin pigmentation in the vegetative tissue tassel and leaf sheaths but not in the seeds. The VMLs show an inverse pattern of anthocyanin pigmentation compared to its CML parent.
2

## Slide 14
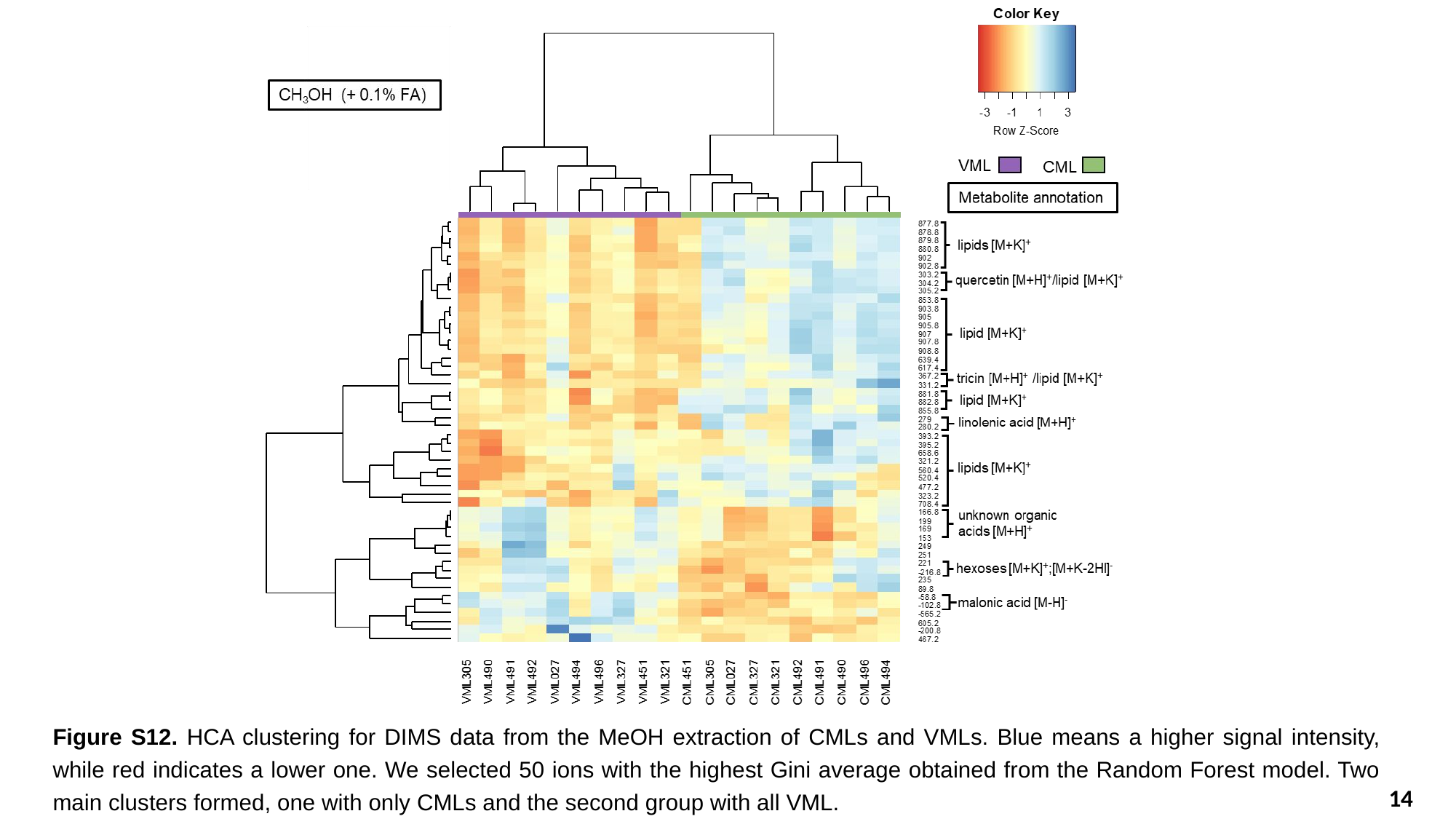

Figure S12. HCA clustering for DIMS data from the MeOH extraction of CMLs and VMLs. Blue means a higher signal intensity, while red indicates a lower one. We selected 50 ions with the highest Gini average obtained from the Random Forest model. Two main clusters formed, one with only CMLs and the second group with all VML.
2

## Slide 15
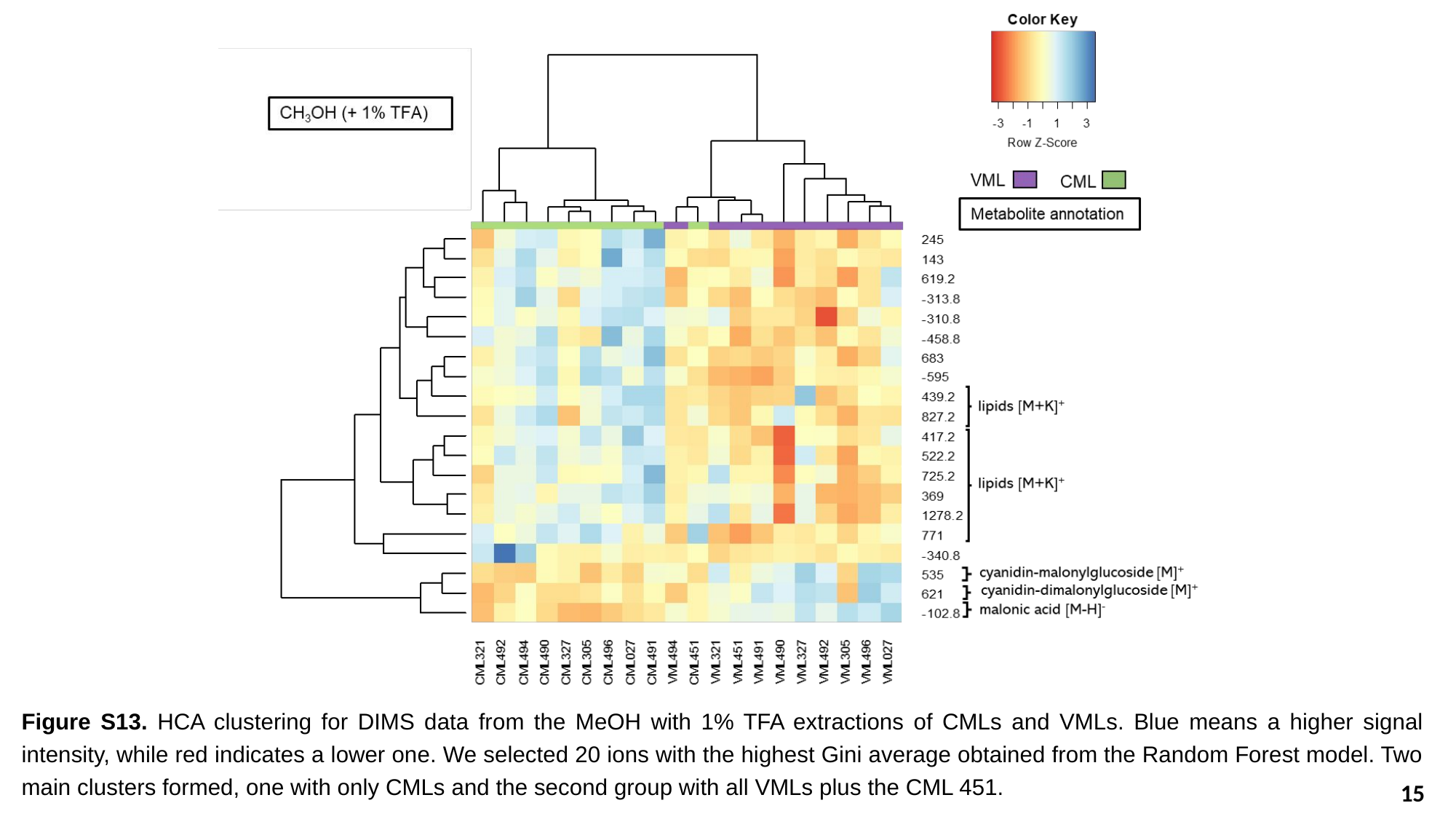

Figure S13. HCA clustering for DIMS data from the MeOH with 1% TFA extractions of CMLs and VMLs. Blue means a higher signal intensity, while red indicates a lower one. We selected 20 ions with the highest Gini average obtained from the Random Forest model. Two main clusters formed, one with only CMLs and the second group with all VMLs plus the CML 451.
<number>

## Slide 16
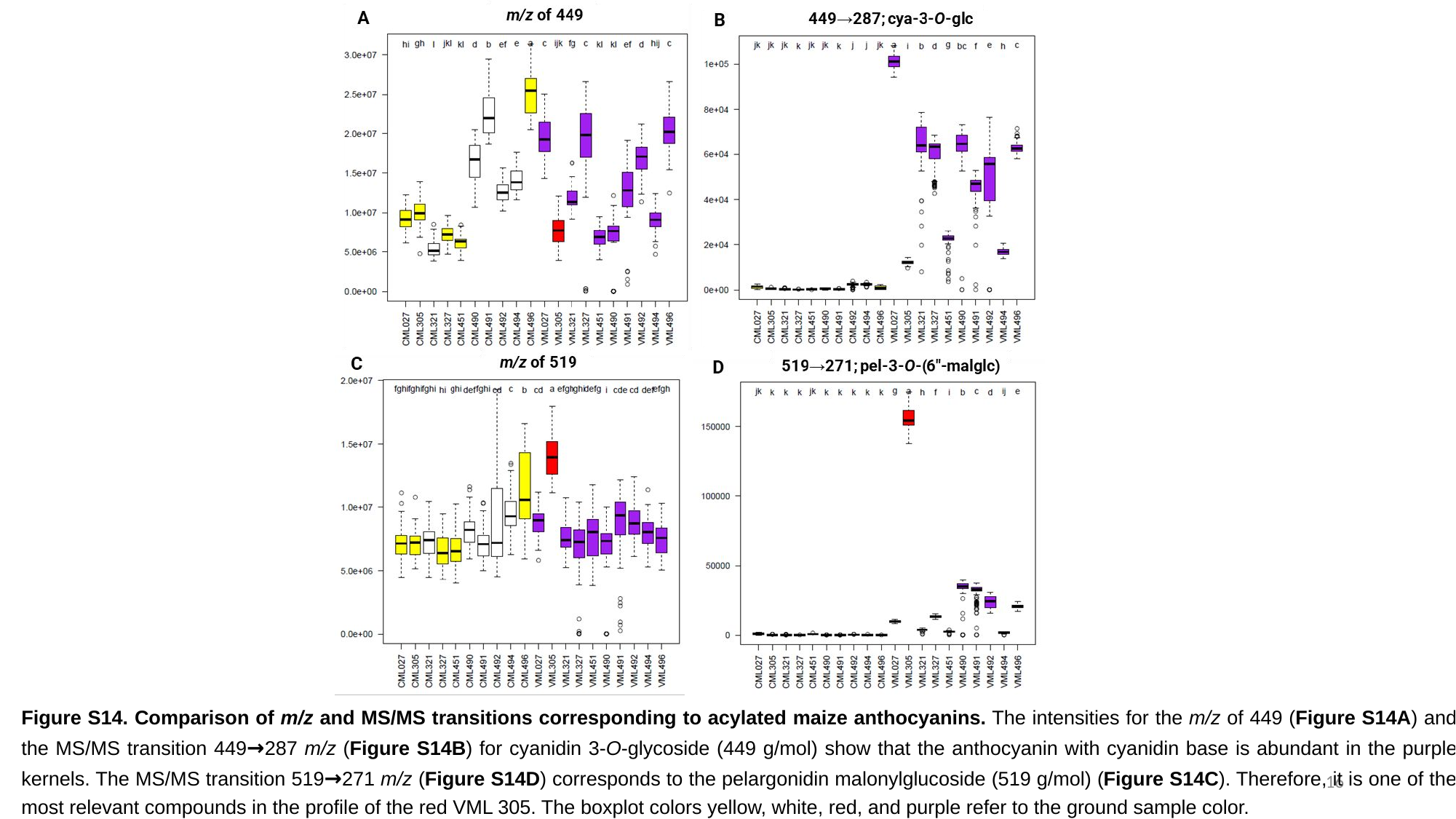

Figure S14. Comparison of m/z and MS/MS transitions corresponding to acylated maize anthocyanins. The intensities for the m/z of 449 (Figure S14A) and the MS/MS transition 449→287 m/z (Figure S14B) for cyanidin 3-O-glycoside (449 g/mol) show that the anthocyanin with cyanidin base is abundant in the purple kernels. The MS/MS transition 519→271 m/z (Figure S14D) corresponds to the pelargonidin malonylglucoside (519 g/mol) (Figure S14C). Therefore, it is one of the most relevant compounds in the profile of the red VML 305. The boxplot colors yellow, white, red, and purple refer to the ground sample color.
16
